# Assessment of Pollen Viability for Wheat

**DOI:** 10.3389/fpls.2019.01588

**Published:** 2020-01-22

**Authors:** Daniela Impe, Janka Reitz, Claudia Köpnick, Hardy Rolletschek, Andreas Börner, Angelika Senula, Manuela Nagel

**Affiliations:** ^1^Genebank Department, Leibniz Institute of Plant Genetics and Crop Plant Research (IPK), Seeland, Germany; ^2^Department of Molecular Genetics, Leibniz Institute of Plant Genetics and Crop Plant Research (IPK), Seeland, Germany

**Keywords:** *in vitro* pollen germination, raffinose, recalcitrant pollen, impedance flow cytometry, fluorescein diacetate staining, stigmatic germination

## Abstract

Wheat sheds tricellular short-lived pollen at maturity. The identification of viable pollen required for high seed set is important for breeders and conservators. The present study aims to evaluate and improve pollen viability tests and to identify factors influencing viability of pollen. In fresh wheat pollen, sucrose was the most abundant soluble sugar (90%). Raffinose was present in minor amounts. However, the analyses of pollen tube growth on 112 liquid and 45 solid media revealed that solid medium with 594 mM raffinose, 0.81 mM H_3_BO_3_, 2.04 mM CaCl_2_ at pH5.8 showed highest pollen germination. Partly or complete substitution of raffinose by sucrose, maltose, or sorbitol reduced *in vitro* germination of the pollen assuming a higher metabolic efficiency or antioxidant activity of raffinose. *In vitro* pollen germination varied between 26 lines (P < 0.001); between winter (15.3 ± 8.5%) and spring types (30.2 ± 13.3%) and was highest for the spring wheat TRI 2443 (50.1 ± 20.0%). Alexander staining failed to discriminate between viable, fresh pollen, and non-viable pollen inactivated by ambient storage for >60 min. Viability of fresh wheat pollen assessed by fluorescein diacetate (FDA) staining and impedance flow (IF) cytometry was 79.2 ± 4.2% and 88.1 ± 2.7%, respectively; and, when non-viable, stored pollen was additionally tested, it correlated at r = 0.54 (P < 0.05) and r = 0.67 (P < 0.001) with *in vitro* germination, respectively. When fresh pollen was used to assess the pollen viability of 19 wheat, 25 rye, 11 barley, and 4 maize lines, correlations were absent and *in vitro* germination was lower for rye (11.7 ± 8.5%), barley (6.8 ± 4.3%), and maize (2.1 ± 1.8%) pollen compared to wheat. Concluding, FDA staining and IF cytometry are used for a range of pollen species, whereas media for *in vitro* pollen germination require specific adaptations; in wheat, a solid medium with raffinose was chosen. On adapted media, the pollen tube growth can be exactly analyzed whereas results achieved by FDA staining and IF cytometry are higher and may overestimate pollen tube growth. Hence, as the exact viability and fertilization potential of a larger pollen batch remains elusive, a combination of pollen viability tests may provide reasonable indications of the ability of pollen to germinate and grow.

## Introduction

Wheat (*Triticum aestivum* L.) is an allohexaploid (2n = 6x = 42, AABBDD), self-pollinating species adapted to a wide range of temperate environments (Shewry, 2009). Annually about 770 million tons of wheat seeds are produced for human diet and agricultural purposes (http://www.fao.org/faostat/en/). Although, there had been significant increases in the global wheat yield development until 1990ies, about 38.8% of the global wheat production area has experienced yield stagnation in the recent years (Ray et al., 2012). Here, hybrid breeding promises an increase in grain yield and stability through the recognition of heterotic pattern (Zhao et al., 2015). Among other factors, successful wheat hybrid breeding relies on the haploid male gametophyte that is shed after second pollen mitosis completed. The mature tricellular wheat pollen has relatively high moisture contents (McCormick, 2004) and is known to be short-lived. Therefore, pollination is necessary within 30 to 40 min after pollen shedding to achieve successful seed sets (Fritz and Lukaszewski, 1989).

Viable pollen is important for species dispersal, fitness, and survival of the next plant generation. It is also essential for directed plant breeding and, consequently, crop improvement. Pollen viability comprises different aspects of pollen performance such as fertilization ability, germinability, and stainability (Dafni and Firmage, 2000). Common techniques to elucidate pollen viability are staining techniques, *in vitro* germination, seed set as well as *in vivo*, and semi-*in situ* germination on the excised stigma, also termed stigmatic germination. In the last two, pollen tube growth toward or on stigmas is observed by contrasting dye and the results are assumed to give most accurate estimations of the seed set (Esser, 1955; Dionne and Spicer, 1958). However, incompatibilities, post-fertilization barriers and limited measurability may restrict the accuracy of these tests (Dafni and Firmage, 2000).

Staining of pollen aims to visualize specific compounds, contents, or cellular compartments related to pollen viability. Potassium iodide, aniline blue, and acetocarmine stain starch, callose, and chromatin, respectively, and the absence of colors indicate non-viable pollen (Stanley and Linskens, 1974). Comparable, the Alexander stain discriminates aborted pollen grains from non-aborted pollen grains. Here, the cytoplasm is colored red, whereas the cell walls are stained green. When the cytoplasm is absent, the green cell walls become visible and indicate a lack of function (Alexander, 1969). In the 1970ies, Heslop-Harrison and Heslop-Harrison (1970) developed a viability test based on the fluorochromatic reaction which included tests of the membrane integrity and enzyme activity. The non-fluorescent polar dye fluorescein diacetate (FDA) passes intact semi-permeable membranes. Intracellular non-specific esterases hydrolyze FDA and the fluorescein accumulates in the cytoplasma which shows a bright green fluorescence. When the plasmalemma is impaired the fluorescein can leave the pollen grains and a uniform background fluorescence can be observed which is an indication that the pollen grains are non-viable. Although staining methods offer the possibility to distinguish aborted and non-aborted fresh pollen, they often fail to discriminate different viability levels (Ge et al., 2011).

Refined viability estimations can be achieved by *in vitro* germination or the impedance flow (IF) cytometry. IF cytometry measures electrical cell properties of single cells using microfluidic chips and has been successfully applied to measure the physiological cell state of bacteria (David et al., 2012). Pollen grains are stimulated by radio frequencies from 0.1 to 30 MHz with alternating current and resulting data are related to cell size, membrane capacitance, cytoplasmic conductivity of single cells, and cell concentration. For mature cucumber, sweet pepper, and tomato pollen, high correlations were found between viability results of IF cytometry and FDA staining (Heidmann et al., 2016). Nevertheless, the potential of pollen to germinate and grow can be best analyzed by pollen tube growth in liquid or on solid media, also termed *in vitro* germination. Brewbaker and Kwack (1963) developed one of the first comprehensive pollen culture media suitable for 86 species. For wheat pollen, Cheng and McComb (1992) achieved maximum pollen tube length on medium containing raffinose. Due to high expenditures for raffinose, Jian et al. (2014) and Jayaprakash et al. (2015) replaced raffinose either by sucrose or maltose, respectively and achieved pollen germination up to 95% (Jayaprakash et al., 2015). However, except of these studies, wheat pollen germination has not been investigated in greater detail up to now. Therefore, the present study aims to compare pollen germination, staining assays, and IF cytometry across different wheat lines to establish a reproducible protocol to assess wheat pollen viability and to elucidate influencing factors. The term “pollen viability” will be used as an umbrella term describing the capacity of pollen to live, grow, germinate, or develop (Dafni and Firmage, 2000). By comparing 157 solid and liquid media, varying in main sugars, H_3_BO_3_, CaCl_2_·2H_2_O/Ca(NO_3_)_2_·2H_2_O concentration, pH, and other components, we selected a raffinose based medium triggering the highest *in vitro* germination. The results were compared with the pollen viability assessed by semi *in vivo* germination on the stigma, termed stigmatic germination, FDA staining, Alexander staining, and IF cytometry in various wheat lines. To test the suitability of the medium for other species of the Poaceae family, pollen of different rye, barley, and maize lines were germinated and results are discussed in relation to the required energy source for pollen germination, the effect of the line/species, and environment.

## Material and Methods

### Plant Material and Pollen Extraction

Seeds of 26 wheat, 25 rye (*Secale cereale* L.), 11 barley (*Hordeum vulgare* L.), and 4 maize (*Zea mays* L.) lines comprising listed varieties, breeding lines, and accessions were used for pollen viability assessments. Seeds were provided by the Federal *Ex situ* Gene Bank at IPK Gatersleben (https://doi.org/10.5447/ipk/2019/9), Germany and from the Maize Genetics Stock Center (http://maizecoop.cropsci.uiuc.edu) (**Supplementary Table S1**) and germinated in a standard culture medium (Substrate1, Klasmann-Deilmann GmbH, Geeste, Germany) at 20 ± 2°C. One-week old seedlings of wheat, rye, and barley lines were subjected to 4 ± 1°C for four (spring types) and six weeks (winter types). Vernalized plants were transferred into pots containing a sand/soil mixture (70% compost soil, 20% white peat, 10% sand) and grown under optimum conditions (regular watering and fertilization, 16 h light) at 20 ± 2°C in the greenhouse. Maize seeds were germinated in 0.25 L pots at 22/20°C (day/night) and 13 h light. After 3 weeks, plantlets were transferred to 20 L pots and developed under optimum conditions (regular watering and fertilization) at 25/20°C (day/night) and 16 h light. In total, seeds of 19 wheat, 25 rye, and 11 barley lines were sown in two cultivation periods in August (1^st^ set) and September (2^nd^ set) in 2017 and mature pollen was available between November and January (1^st^ set) and between December and March (2^nd^ set). At the beginning of anthesis, spikes were cut between 8:00 and 10:00 a.m., kept in water and used within 6 h.

**Figure 1:**
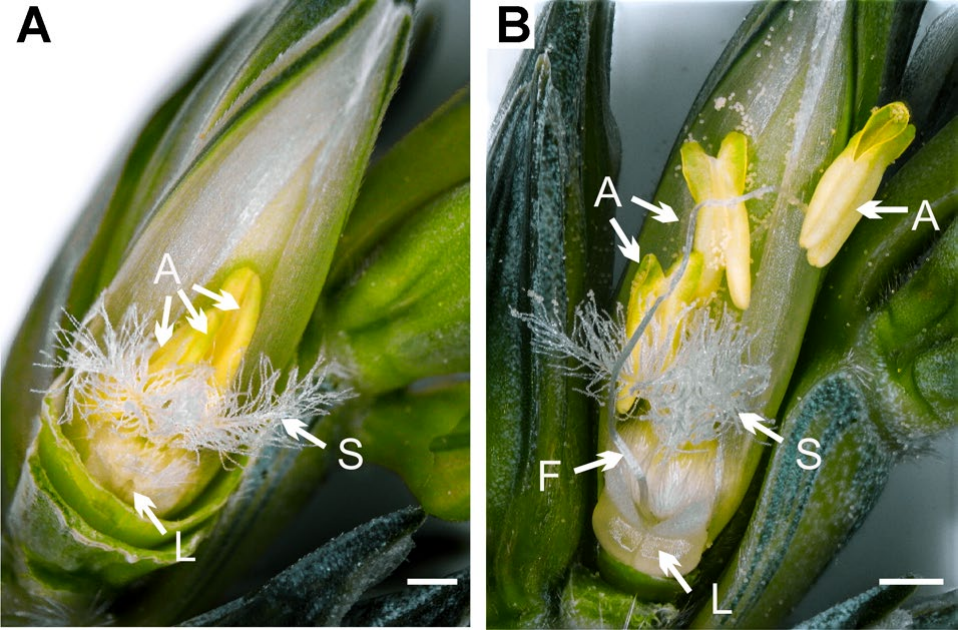
Structural changes in wheat florets before and after pollen shedding. **(A)** Before pollen shedding, filaments were short, anthers (A) closed, greenish yellow, and showed fleshy anther lobes. To support the pollen extraction and maturation, awns, glumes, and lemmas were removed. **(B)** Ten minutes after removing the glumes, lodicules (L) swelled, filaments (F) elongated, anther tips opened, and mature pollen shed on the stigma (S). At this stage, the maximum pollen maturity was reached and pollen was used for all experiments. Scale bars = 1 mm.

Only mature pollen was used for all experiments. To stimulate pollen maturation, awns, glumes, and lemmas were carefully removed and pollen was sampled when lodicules swelled, the stigma fanned out, filaments elongated, and anthers enlarged and turned greenish to bright yellow (**Figure 1**, **Supplementary Video S1**). Before the tip of the anther opened, at minimum three anthers were taken and pollen shedding was supported by opening gently with a needle. For some experiments, non-viable pollen was required. According to Fritz and Lukaszewski (1989), wheat pollen loses germination at ambient conditions within 45 min. Therefore, to achieve non-viable pollen, we stored the wheat pollen at ambient conditions (50.0% relative humidity and 23°C) for >60 min. For each replicate, anthers of different plants were used. Due to variations in pollen production, lines varied between some experiments.

### Modification and Evaluation of Liquid Pollen Growth Media

To assess pollen tube growth, liquid media based on Cheng and McComb (1992); Jayaprakash et al. (2015), and Jian et al. (2014) were modified in contents of CaCl_2_·2H_2_O/Ca(NO_3_)_2_·2H_2_O, H_3_BO_3_ and the effect of different sugars and pH values was evaluated. Media were prepared using between 300 and 900 mM of sucrose, maltose, and raffinose and pH was adjusted to 5.8 and 7.3 (**Supplementary Table S2**). The media according to Jayaprakash et al. (2015) and Jian et al. (2014) contained 0.81 mM MgSO_4_·7H_2_O and 0.99 mM KNO_3_ in addition.

By using the sitting drop technique (Shivanna and Rangaswamy, 1992), 10 to 20 µl of the liquid media were placed in the wells of microscopic slides. Spikes of the lines ‘Ferrum’, ‘Piko’, and ‘Hermann’ were harvested and for each of the three replicates, pollen was pooled from mature anthers of one single spike. Pollen were shed on the droplets (without coverslip to avoid hypoxia) and further development was observed under the microscope immediately (Eclipse LV100, Nikon, Tokyo, Japan) (Stanley and Linskens, 1974). Due to rapid crystallization of the sugars, the detailed count of pollen tubes was not possible. Therefore, the frequency of pollen tubes and pollen bursting, and pollen tube lengths was estimated for a total of about 500 pollen grains located in ten microscopic fields (**Supplementary Figure S1**). Low, medium, and high frequency of pollen tubes refers to 1, up to 10 and more than 10 pollen grains, respectively, that developed pollen tubes. Low, medium, high frequency of pollen bursting refers to one, half of the pollen batch and most pollen that burst, respectively. Pollen tubes were defined as short, medium, and long when pollen tubes were shorter, between the one- and twofold or longer than the twofold diameter of the pollen, respectively.

### Modification and Evaluation of Solid Pollen Growth Media

Solid media were assessed in three steps. 1^st^) Liquid media (**Supplementary Table S2**) according to Cheng and McComb (1992) and Jayaprakash et al. (2015) were solidified using 0.3% Gelrite and pH adjusted at 5.8 (**Supplementary Table S3**). Both media were further modified using ε-aminocaproic acid (EACA), peptone water (Pep), PEG 4000, PEG 8000, and different concentrations of H_3_BO_3_, CaCl_2_·2H_2_O/Ca(NO_3_)_2_·2H_2_O, maltose, sucrose, and raffinose. All media were used to test pollen germination of the lines ‘Ferrum’, ‘Piko’, and ‘Hermann’. 2^nd^) A solid medium containing 0.81 mM H_3_BO_3_, 2.04 mM CaCl_2_·2H_2_O, and 594 mM raffinose at 5.8 pH stimulated highest pollen germination in the 1^st^ step and was termed “Basic” medium. To test the effect of different factors such as lines (‘Ferrum’, ‘Dialog’, ‘Einstein’, and TRI 9102), macronutrients (sucrose, maltose, mannitol, sorbitol), additives (H_3_BO_3_, EACA, IAA, GA_3_) and concentrations, the “Basic” medium was further modified (**Table 1**) and the osmolality measured using a vapor pressure osmometer (Vapro 5520, Wescor, Langenfeld, Germany). 3^rd^) The “Basic” medium showed best results in the 2^nd^ step and was consequently used for comparisons of pollen germination of 19 wheat, 25 rye, 11 barley, and 4 maize lines. All media were autoclaved for 20 min and stored at 5°C in sealed petri-dishes until usage.

**Table 1:** Different modifications and osmolality of the solid media “Basic”.

Media	H_3_BO_3_	CaCl_2_ · 2H_2_O	Sucrose	Maltose	Raffinose	EACA	Pep	Mannitol	Sorbitol	IAA	GA_3_	Osmolality
	mM	mM	mM	mM	mM	mg L^−1^	mg L^−1^	mM	mM	mM	mM	mmol kg^−1^
Basic	0.81	2.04			594							700
EACA + Pep	0.81	2.04			594	500	100					865
EACA	0.81	2.04			594	500						864
Pep	0.81	2.04			594		100					767
High CaCl_2_	0.81	3.04			594	500	100					887
High H_3_BO_3_	1.62	2.04			594	500	100					818
Sucrose	0.81	2.04	292		396	500	100					649
Maltose	0.81	2.04		292	396	500	100					644
Mannitol	0.81	2.04			594	500	100	300				970
Sorbitol	0.81	2.04			594	500	100		300			2465
IAA	0.81	2.04			594	500	100			0.01		934
GA_3_	0.81	2.04			594	500	100				0.01	862

ε-Aminocaproic acid (EACA), CaCl_2_ · 2H_2_O (CaCl_2_), peptone water (Pep), mannitol, sorbitol, gibberellic acid (GA_3_), or indole-3-acetic acid (IAA) were added to the “Basic” medium or raffinose was partly substituted by sucrose or maltose. Sugar concentrations of 292, 396, and 594 mM correspond to 10, 20, and 30%, respectively.

**Figure 2:**
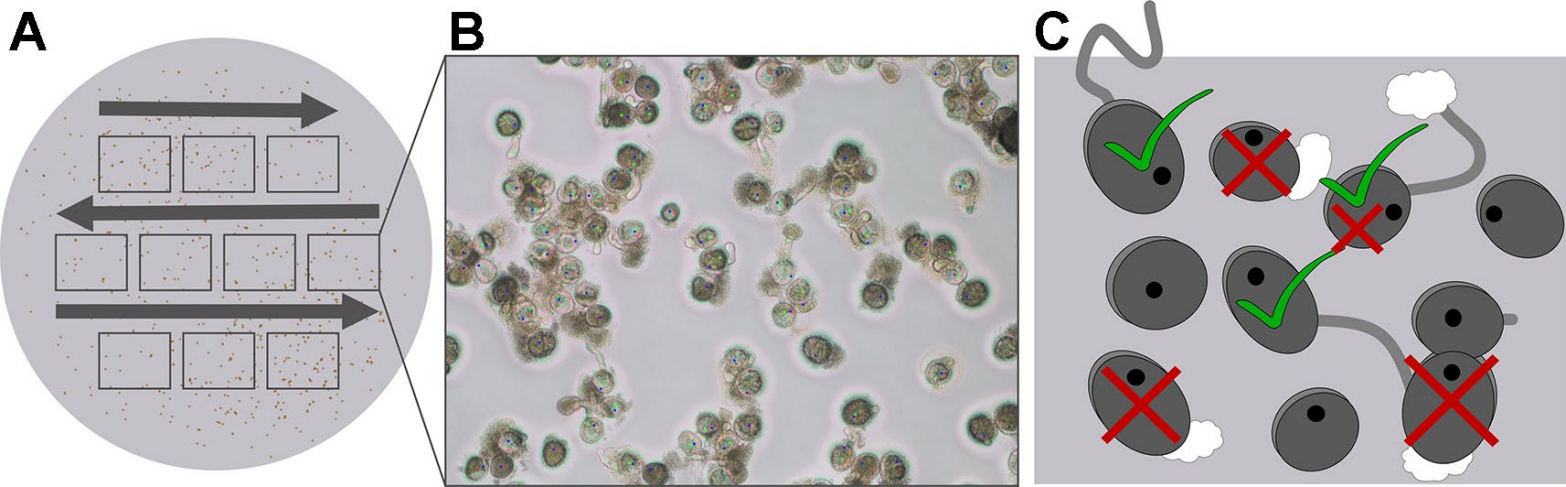
Evaluation of pollen germination on solid media. **(A)** Pollen was distributed on solid medium and 10 independent pictures (rectangles) were taken for further evaluation. **(B)** One of 10 pictures taken for pollen germination analysis is shown. **(C)** Each picture was evaluated and the total number of pollen (black dots), germinated pollen (green ticks), and burst pollen (red crosses) counted.

For all solid media, three replicates were prepared and each replicate represented mature pollen from anthers of a single spike. The pollen was evenly dusted on the medium. To ensure an optimal pollen tube development, petri-dishes were placed without lid in a chamber and kept at 98.0 ± 0.5% relative humidity and 23°C for 30 min. Pollen germination was observed for each petri-dish by taking 10 non-overlapping pictures (Eclipse LV100, Nikon, Tokyo, Japan) at 100 x magnification (**Figure 2**). The total number of pollen, mostly between 500 and 1,000 pollen grains, burst pollen, and pollen tubes that exceeded the lengths of the pollen radius were counted manually using the software NIS elements v. 4.11 (Nikon Metrology, Brighton, USA) (Richter and Powles, 1993; Burke et al., 2007; Cerović et al., 2014). Shrunken or sterile pollen was not considered. After counting, the percentage of germinated and burst pollen was calculated.

### Acetocarmine Staining to Identify Stigmatic Pollen Germination

*In vitro* pollen germination of the wheat line TRI 9102 was compared to stigmatic germination. Before anthesis, inner florets of wheat spikes were discarded and, in each spikelet, the two remaining immature florets were emasculated. After 2 days, wheat spikes were cut between 8:00 and 10:00 a.m., kept in water, and used within 6 h. Pollen was shed over fanned stigmas and germinated for about 30 min. For better visualization, stigmas were placed in a droplet of acetocarmine (Morphisto, Frankfurt, Germany) and kept in a chamber above filter paper soaked in 45% acetic acid for another 30 min. The stigmas were washed in 45% acetic acid and the ovaries carefully removed from the stigma. After washing, stigmas were placed into a drop of 45% acetic acid on a microscopic slide, covered using a coverslip, and germinated pollen was counted as previously described (**Figure 2**). Stigmatic germination was tested on 14 stigmas obtained from different plants (n = 14). In total, >70 fresh pollen grains and about 20 pollen grains stored at ambient conditions for >60 min were dusted on each stigma. Stigmatic pollen germination was compared to *in vitro* germination which was analyzed in 14 biological replicates (n = 14) using >300 fresh pollen and >300 stored pollen each.

### Fluorescein Diacetate Staining and Alexander Staining to Test Pollen Viability

*In vitro* pollen germination was compared to FDA staining (Heslop-Harrison and Heslop-Harrison, 1970) and Alexander staining (Alexander, 1969). Five biological replicates (n = 5) of about 300 to 600 pollen grains each were analyzed for the lines ‘Ferrum’, ‘Hermann’, and TRI 9102 each. FDA stain was prepared freshly before usage. Shortly, 2.4 mM FDA dissolved in acetone was mixed with 0.5 *M* sucrose solution until the solution turned milky (Pinillos and Cuevas, 2008). Fresh pollen was shed on a droplet containing FDA and pollen-FDA-solution was mixed. Due to the bursting of the pollen, the stain fainted fast into the drop. Bright green fluorescing pollen was observed using UV light and a fluorescence microscope (Axiolab, Zeiss, Jena, Germany) and counted as stainable (**Supplementary Figures S2A, B**).

Alexander stain was prepared according to Alexander (1987). Again, fresh and stored wheat pollen grains were shed on a droplet of the Alexander stain placed on a microscopic slide and closed by a coverslip. Pollen stained magenta was considered intact, whereas blue-green pollen was considered to be non-viable or sterile (**Supplementary Figures S2C, D**).

### Impedance Flow Cytometry

Pollen viability of the lines ‘Ferrum’, ‘Hermann’, and TRI 9102 was tested using IF cytometry and five replicates (n = 5) each. Briefly, for each replicate, between 500 and 1,500 of fresh pollen or non-viable, stored pollen grains were transferred into 1 mL IF cytometry measurement buffer (AF6, Amphasys, Lucerne, Switzerland), filtered using 100 µm pore size and loaded onto a channel chip (channel size 120 µm) inserted in the IF cytometer type Ampha Z32 (Amphasys, Lucerne, Switzerland). Measurements were carried out at 1 MHz at the default settings for wheat pollen (trigger level 0.1 V, frequency 1, modulation 3, amplifier 6, and demodulation 1). Data of 1.4 x 10^3^ ± 640 cells per sample using a concentration between 250 and 5.5 x 10^3^ cells mL^−1^ were collected and analyzed using AmphaSoft v 2.0 version (Amphasys, Lucerne, Switzerland) (**Supplementary Figures S2E, F**).

### Sugar Analysis

Soluble sugars were extracted from fresh and stored pollen samples (1.5 ± 0.8 mg) of the lines ‘Ferrum’, ‘Hermann’, and TRI 9102 using n = 4 biological replicates each. Pollen was mixed with 0.2 mL ethanol (60% v/v) and stored at −20°C until further analysis (Rolletschek et al., 2011). For efficient extraction, the samples were sonicated for 5 min. After centrifugation (5 min at 13,000 rpm) the supernatants were diluted with 10% methanol (1:50 v/v) and used directly for sugar analysis using ion-exchange chromatography coupled to pulsed amperometric detection (ICS-3000; Thermo Fisher). Chromatographic separation was carried out on a PA1 column (2 × 250 mm) and a PA1 guard column (2 × 50 mm) at 25°C by applying an isocratic run with 400 mM NaOH at a constant flow rate of 0.7 mL min^−1^ over 5 min. A mixture of glucose, fructose, sucrose, raffinose, stachyose, and verbascose was used as authentic standards and for external calibration. The identification of sugars was performed by comparing retention times of individual sugars in the reference *vs.* analyzed solution, and quantification was based on peak areas. Due to the low availability of pollen, the dry weight could not be determined. Therefore, the change in weight was measured on a second set of ‘Ferrum’ and TRI 9102 at a later time point. The fresh weight of fresh pollen was corrected by 0.39 and the fresh weight of stored pollen was corrected by 0.91 to show the data on the content of individual sugars on a dry weight bases.

### Statistical Analysis

Statistical analysis was conducted using SigmaStat 4.0 (Systat Software, 2016). All data were tested for normal distribution using the Shapiro-Wilk test. GenStat 18 (VSN International Ltd, 2016) was used to conduct Spearman correlation analysis at P < 0.05 and analysis of variance (ANOVA). Statistical differences were evaluated using the least significant difference at P < 0.05 (LSD5%). If the normality test failed, a balanced design was used for ANOVA and pairwise multiple comparisons using Holm-Sidak method was chosen.

## Results

### Solid Medium Containing Raffinose Is Suitable for the Assessment of *In Vitro* Pollen Germination

Pollen tube growth was mostly stimulated when raffinose was available in liquid media. Of the 112 liquid media that were based on different protocols (Cheng and McComb, 1992; Jian et al., 2014; Jayaprakash et al., 2015) and differed in sugar composition (sucrose, maltose, raffinose) and in pH (pH 5.8, pH 7.3), pollen tube growth was visible in 25 liquid media. The highest number of germinated pollen in liquid media was found in media developed by Cheng and McComb (1992) containing raffinose (**Supplementary Table S2**). Here, the concentrations of H_3_BO_3_ and CaCl_2_·2H_2_O affected significantly the pollen tube development. Reducing the H_3_BO_3_ concentration from 1.62 to 0.81 mM decreased the number of pollen showing pollen tubes, whereby the reduction of CaCl_2_·2H_2_O from 2.04 to 1.02 mM resulted in a higher number of germinated pollen. When raffinose was partly substituted by sucrose or maltose a low frequency of pollen tube development was observed in two media with a pH adjusted to 5.8, each. However, liquid media were difficult to handle due to different focal planes, uneven distribution of pollen grains and rapid crystallization of sugars (**Supplementary Figure S1**). Therefore, in subsequent experiments selected liquid media, resulting in the highest pollen germination, were solidified and used for further germination tests.

**Figure 3:**
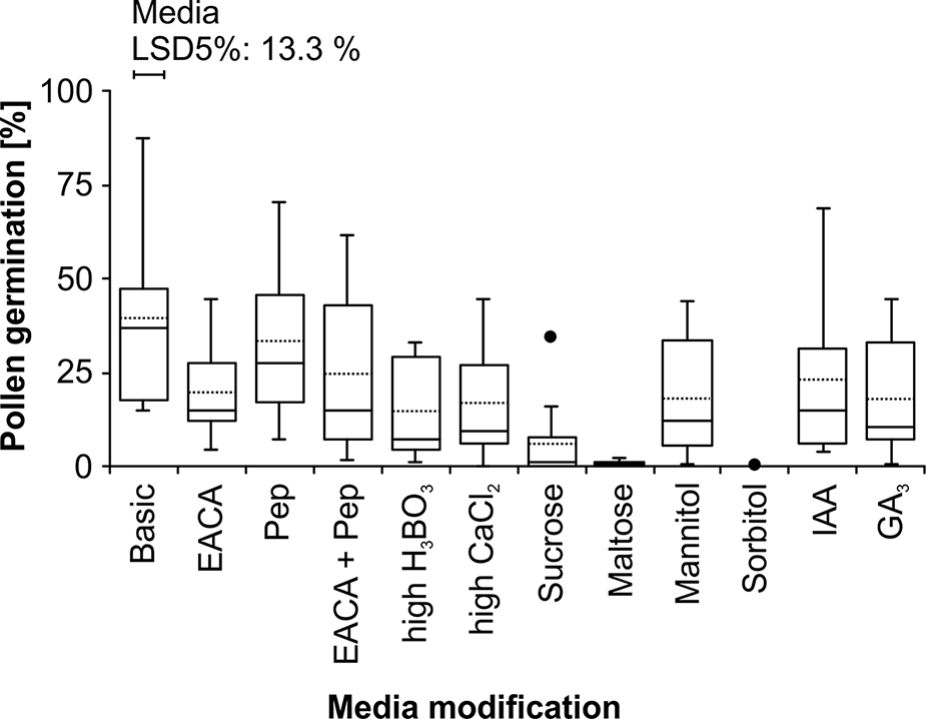
Wheat pollen germination depends on media composition. The ‘Basic’ pollen medium was modified according to **Table 1** and pollen of wheat lines ‘Ferrum’, ‘Einstein’, ‘Dialog’, and TRI 9102 (n = 3 for each line, >805 pollen for each replicate) were analyzed for pollen tube development (**Figure 2**). Box plots represent means (dashed line), medians (solid line), lower and upper quartiles; whiskers show the 98^th^ percentile; and dots show outliers. Significant differences between the means of the media are indicated by the least significant differences at P < 0.05 (LSD5%). CaCl_2_, calcium chloride dihydrate (CaCl_2_·2H_2_O); EACA, ε-aminocaproic acid; GA_3_, gibberellic acid; IAA, indole-3-acetic acid; Pep, peptone water; H_3_BO_3_, boric acid.

Pollen germination could be quantified on solid media containing raffinose (**Figure 3**, **Supplementary Table S3**). Of in total 34 solid media, *in vitro* pollen germination was only observed on two media prepared according to Cheng and McComb (1992). On the average of four different lines, germination was 13.3 ± 10.4% in a media containing 1.62 mM H_3_BO_3_, 1.02 mM CaCl_2_·2H_2_O, and 594 mM raffinose and 32.7 ± 14.2% in a media containing 0.81 mM H_3_BO_3_, 2.04 mM CaCl_2_·2H_2_O, and 594 mM raffinose. However, in both media about 65% of the pollen burst. In solidified media according to Jayaprakash et al. (2015), pollen tube growth was not detected (**Supplementary Table S3**). Therefore, the medium adapted from Cheng and McComb (1992) containing 0.81 mM H_3_BO_3_, 2.04 mM CaCl_2_·2H_2_O, and 594 mM raffinose at 5.8 pH was further modified and the effect of different plant hormones (GA_3_ and IAA), sugars (sucrose and maltose), sugar alcohols (mannitol and sorbitol), and additives (Pep, EACA) was investigated in detail (**Figure 3**). The osmolality of the different media ranged between 644 mmol kg^−1^ (sucrose) and 2,465 mmol kg^−1^ (sorbitol, **Table 1**). Among the four different lines, variations in pollen germination were observed, but this was not related to the osmolality of the solid media. Maltose, sorbitol, and sucrose inhibited most pronouncedly pollen tube development. The “Basic” medium showed highest pollen germination (39.7 ± 25.0%) and, therefore, was chosen for comparisons between different pollen viability tests, lines, and species.

### Stigmatic Pollen Germination Was Higher Compared to *In Vitro* Germination

**Figure 4:**
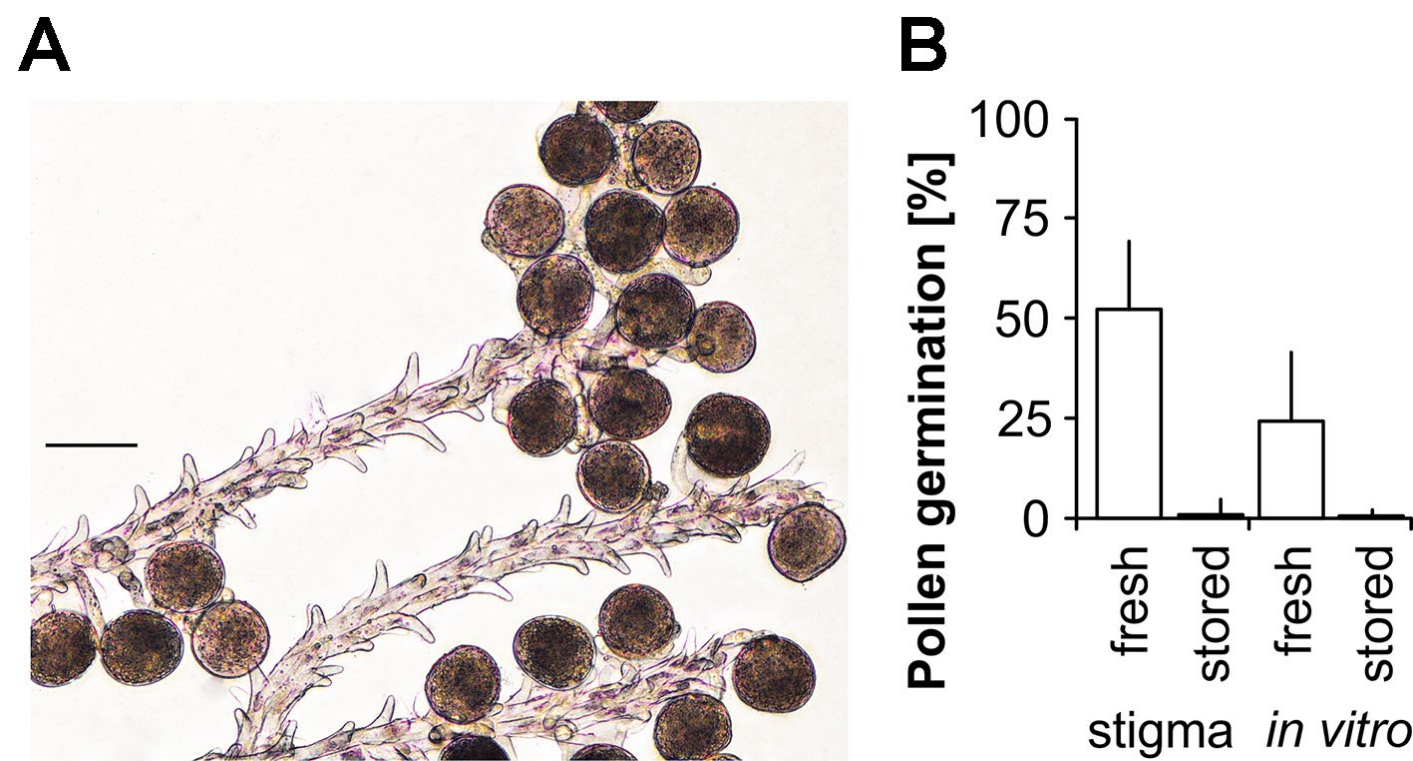
Improved wheat pollen tube growth in the presence of the stigma. **(A)** Pollen tubes grew toward stigma. The selected image section represents an ideal situation of pollen tube growth. **(B)** Comparison of stigmatic pollen germination and *in vitro* germination on medium using fresh pollen and pollen stored at ambient conditions for > 60 min. Mean and standard deviation of n = 14 replicates using >70 pollen each are shown for the line TRI 9102. The least significant differences at P < 0.05 (LSD5%) was estimated at 10.0%. Scale bar = 50 µm.

Wheat stigmas of emasculated florets stimulated pollen tube development of fresh pollen. In total, 52.4 ± 17.0% of the freshly harvested and mature pollen developed pollen tubes and grew toward or entered the stigma (**Figure 4**). A significantly (P < 0.001) lower germination (24.2 ± 17.2%) was observed for pollen grains on the “Basic” medium. In comparison, pollen stored at ambient conditions for 60 min revealed 1.8 ± 3.4 and 0.7. ± 2.4% for stigmatic and *in vitro* germination, respectively. However, the evaluation of stigmatic pollen germination was difficult due to chaotic pollen tube growth and the analysis of different focal layers in the microscope. Furthermore, the number of pollen grains for each stigma is limited and standardizations are hardly possible.

### Analysis of *In Vitro* Germination, Fluorescein Diacetate Staining, Impedance Flow Cytometry, and Soluble Sugars to Assess Wheat Pollen Viability

**Figure 5:**
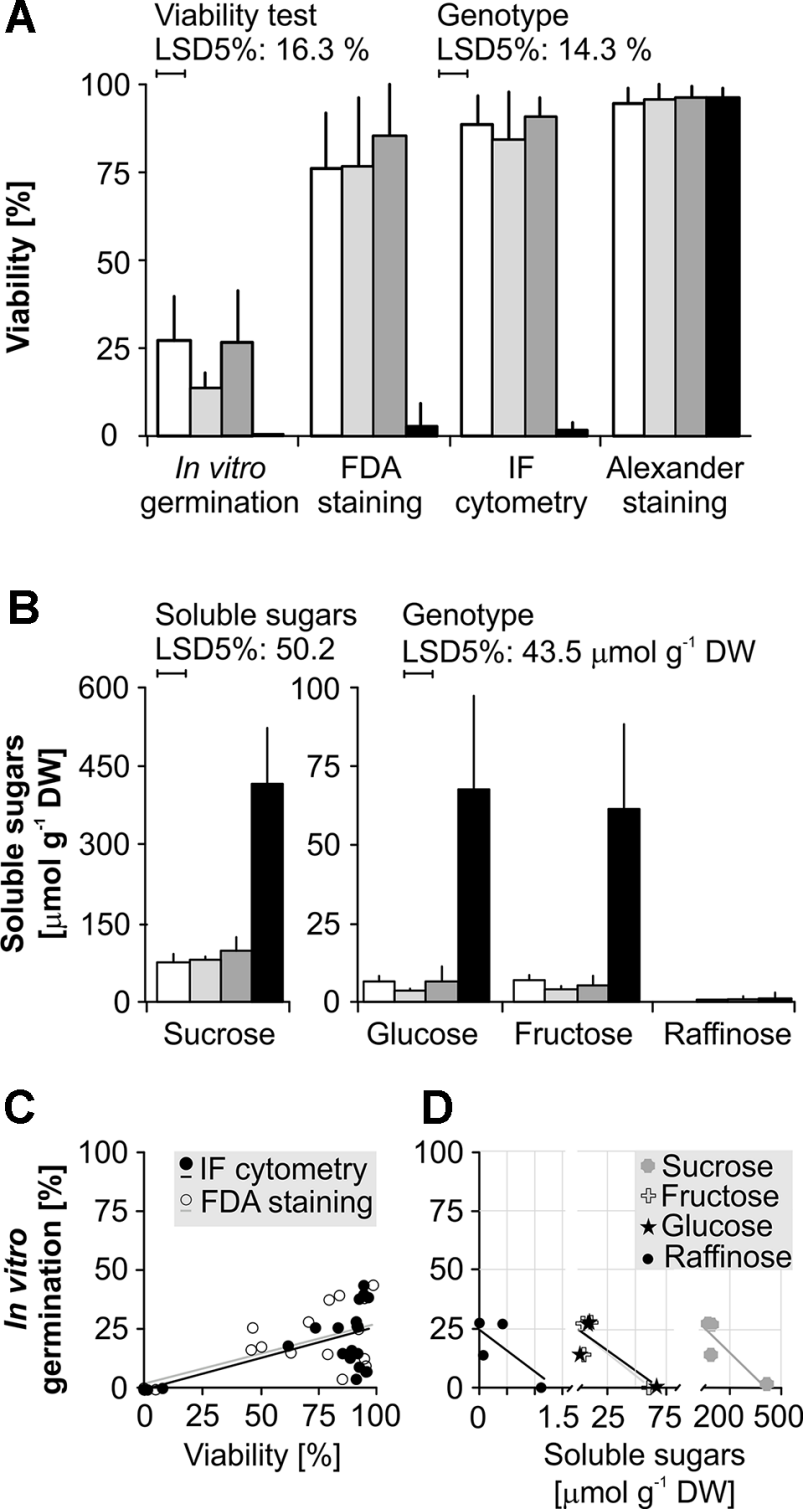
Assessment of wheat pollen viability using physiological, biochemical and physical tests. **(A)** Pollen viability was evaluated for fresh and stored pollen using *in vitro* germination, fluorescein diacetate (FDA) staining, impedance flow (IF) cytometry, and Alexander staining (**Supplementary Figure S2**). Means and standard deviations of n = 5 replicates of fresh pollen are shown for the lines “Ferrum” (white bar), “Hermann” (light gray bar), and TRI 9102 (dark gray bar). The mean and standard deviation of the three lines is shown for stored pollen (black bars) kept under ambient condition for >60 min. **(B)** Soluble sugars were measured in pollen collected immediately after pollen shedding and in pollen stored under ambient conditions for >60 min. Mean and standard deviations of n = 5 replicates are shown for the lines mentioned above and for stored pollen (black bar). **(C)** Relationship between *in vitro* pollen germination and viability assessed by IF cytometry (r = 0.67, P < 0.001) and FDA staining (r = 0.54, P < 0.05) and **(D)** concentration of raffinose (r = −0.29, P = 0.175), glucose (r = −0.72, P < 0.001), fructose (r = −0.73, P < 0.001), and sucrose (r = −0.77, P < 0.001). Significant differences between the means of the viability assays, soluble sugars, and lines are indicated by the least significant differences at P < 0.05 (LSD5%).

Results of wheat pollen viability differed drastically in dependency of the assay (**Figure 5A**). In three wheat lines, percentage of *in vitro* pollen germination was compared with pollen stainability using FDA and Alexander staining and pollen viability after IF cytometry and were on the average 11.5 ± 14.5%, 40.2 ± 41.6%, 96.6 ± 3.4%, and 44.9 ± 44.4%, respectively. These results include the analysis of pollen stored under ambient conditions for >60 min, which was 0.3 ± 0.1%, 1.3 ± 4.2%, 97.7 ± 2.0%, and 1.6 ± 2.2%, respectively, and indicate that *in vitro* germination, FDA staining, and IF cytometry can discriminate between fresh and stored pollen (**Figure 5A**, **Supplementary Figure S2**). Alexander staining showed high viability results for all, fresh, and stored pollen in all lines and the results were significantly different (P < 0.05) compared to those obtained by other methods. Therefore, this approach was not appropriate for wheat pollen viability assessment and was further excluded from comparisons. Significant correlations were observed between results of *in vitro* pollen germination and IF cytometry (r = 0.67, P < 0.001), and FDA staining (r = 0.54, P < 0.05) (**Figure 5C**). However, due to large differences between the results of *in vitro* germination, FDA staining, and IF cytometry, it can be hypothesized that the medium may still not sufficiently reflect the conditions which wheat pollen require for germination.

Sucrose was the major sugar in fresh wheat pollen collected after pollen shedding (**Figure 5B**). Sucrose concentration was at 74.5 ± 16.7 µmol g ^−1^ DW, 96.5 ± 26.4 µmol g ^−1^ DW, and 79.3 ± 6.8 µmol g ^−1^ DW and accounted for 84.5%, 88.7%, and 91.0% of the total amount of soluble sugars for ‘Ferrum’, ‘Hermann’, and TRI 9102, respectively. The content of sucrose was significantly higher (P < 0.05) in comparison to glucose, fructose, and raffinose representing 6.0%, 5.6%, and 0.4%, respectively, of the total amount of soluble sugars across the three lines. Raffinose was not detected in pollen of the line ‘Ferrum’, and stachyose, maltose, and verbascose in any line. Significant higher (P < 0.001) contents of sucrose, glucose, and fructose were found in stored wheat pollen resulting in significant negative correlations between results of *in vitro* pollen germination and sucrose (r = −0.77, P < 0.001), glucose (r = −0.72, P < 0.001), and fructose (r = −0.73, P < 0.001) (**Figure 5D**). The shift in major soluble sugars during viability loss might be a useful indicator for *in vitro* pollen germination.

### Variations in the Response of Different Wheat Varieties to *In Vitro* Germination and Fluorescein Diacetate Staining

**Figure 6:**
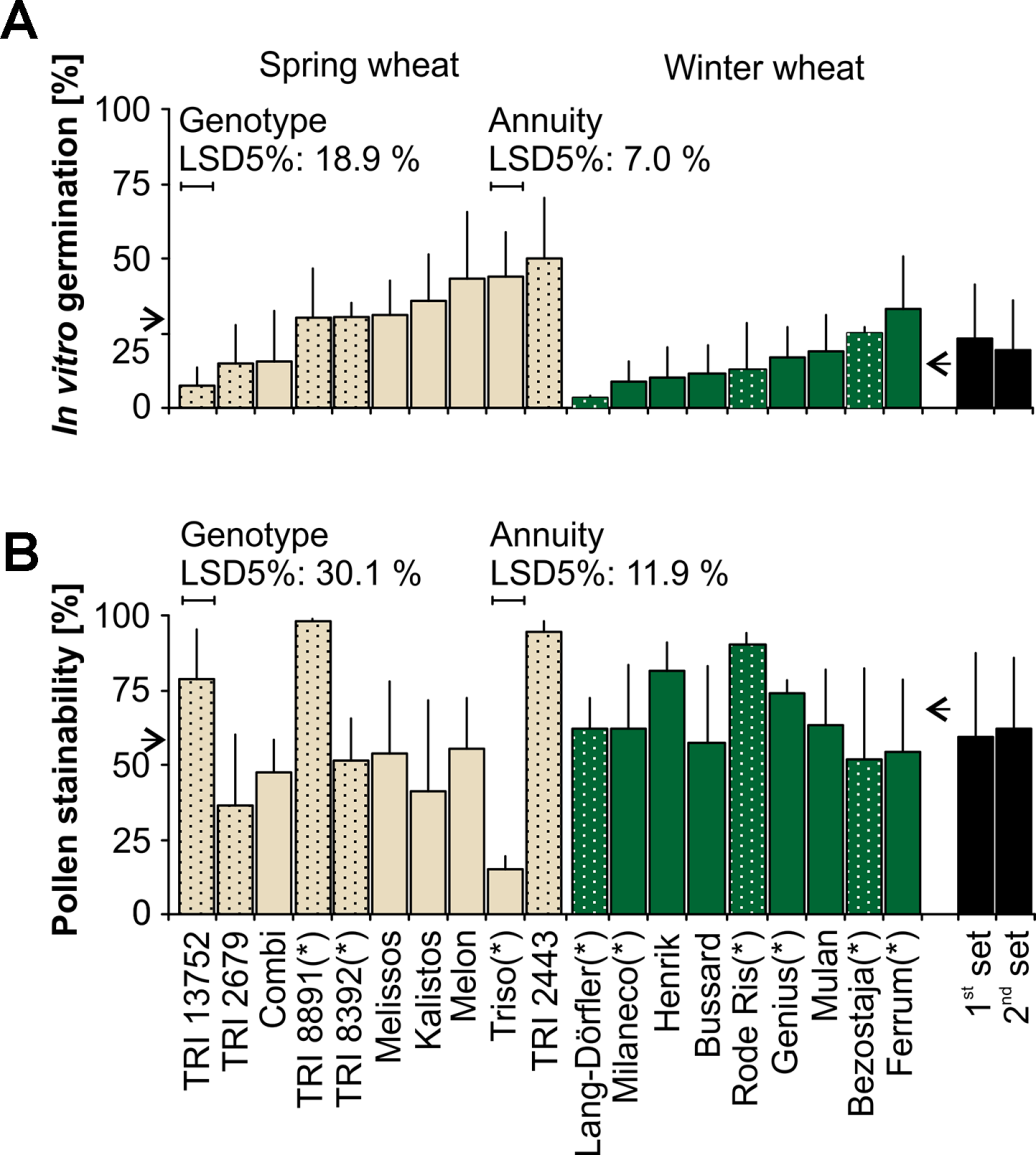
*In vitro* pollen germination and viability assessed by fluorescein diacetate (FDA) staining varied among 19 different wheat lines. **(A)** *In vitro* pollen germination was analyzed for 10 spring and 9 winter wheat lines, among them 7 landraces (spotted bars) and 12 varieties (plain bars). Bars represent means and standard deviations of n = 3 (*) to 6 replicates of 100 to 300 pollen each. **(B)** Pollen stainability was analyzed by FDA staining. Bars represent means and standard deviations of n = 3 (*) to 6 replicates of 100 to 400 pollen each. Arrows indicate mean pollen germination/stainability for spring (left) and winter (right) wheat varieties, respectively. Significant differences between means of lines and spring and winter wheat (annuity) are indicated by the least significant differences at P < 0.05 (LSD5%). Seeds of all lines were grown in two intervals, in August (1^st^ set) and September (2^nd^ set) and mature pollen was available between November and January (1^st^ set) and December and March (2^nd^ set).

*In vitro* pollen germination on medium varied between lines (P < 0.001) and annuity (**Figure 6A**). There was a significant (P < 0.001) difference between winter and spring types showing on the average 15.3 ± 8.5% and 30.2 ± 13.3% pollen germination, respectively. Pollen germination of the spring wheat landrace TRI 13752 and the winter wheat landrace ‘Long-Dörflers Braunweizen’ was lowest with 7.1 ± 6.0% and 3.2 ± 0.7%, respectively. In contrast, pollen of the spring wheat landrace TRI 2443 and the winter wheat variety ‘Ferrum’ germinated at 50.1 ± 20.0% and 32.8 ± 18.1%, respectively. Across the 19 tested lines, higher percentages of pollen were stained by FDA staining (61.7 ± 20.3%) (**Figure 6**) in comparison to the lower percentage of pollen which germinated on the medium (23.1 ± 13.9%). Again, pollen stainability varied between lines (P < 0.001) and pollen of the variety ‘Triso’ showed the lowest germination at 15.0 ± 4.5%. The highest value was obtained for TRI 8891 at 98.3 ± 0.8%. For both, FDA staining and *in vitro* pollen germination, pollen viability was neither associated with biotype (landrace or variety) nor with the sets grown at different time points indicating that variability of pollen viability is influenced by other factors.

### *In Vitro* Germination and Fluorescein Diacetate Staining of Pollen of Different Barley, Rye, and Maize Varieties

Pollen viability varied between lines, species of the Poaceae family and viability assays (**Supplementary Figure S3**). *In vitro* pollen germination ranged between 3.3% and 42.4% for 25 rye lines, between 0.4% and 13.6% for 11 barley lines and between 0.2% and 4.5% for four maize lines. These results were lower in comparison with *in vitro* germination of different wheat lines. Highest variations (<0.001) for pollen stainability assessed by FDA staining were found between 25 rye lines which ranged between 5.4% and 90.8%. Barley and maize lines showed high pollen stainability for all lines and ranged between 81.1% and 97.1%, and 92.7% and 96.3%, respectively. However, correlations between *in vitro* pollen germination and pollen stainability assessed by FDA staining were absent. Comparable to wheat, pollen tube development was not associated with the biotype (landrace or variety) and sets grown at different time points. In summary, *in vitro* pollen germination varied between lines of rye, barley, and maize but was relatively low compared to wheat assuming that the medium for *in vitro* pollen germination may not provide optimal condition required for pollen germination of rye, barley, and maize pollen.

## Discussion

Successful pollination and double fertilization of gametophytes are essential processes for seed production and improvements in plant breeding. Viability assessment of pollen represents an important tool to estimate the physical, biochemical, and biological status and capacity of pollen to generate tubes, penetrate into the stigma, and elongate inside the style until two male gametes are released within female gametophyte. Aside from FDA staining and seed set, *in vitro* germination is seen as a reliable approach to indicate pollen viability but suffers on the development of specific media for different species.

### Sugar Metabolism and Signaling May Determine the Efficiency for Wheat Pollen Germination

Pollen tube growth requires carbohydrates for respiration and elements and energy for cell wall synthesis and transport activities. In wheat pollen, we showed that the soluble carbohydrate sucrose is most abundant and may serve as an important primary energy source in addition to the internal starch reservoirs (Speranza et al., 1997). Supplementary, external carbohydrates such as sucrose and hexoses are required and actively uptaken from the apoplast of the transmitting tissue (Goetz et al., 2017). However, in anthers, high levels of sucrose and the lack/low levels of glucose and fructose can indicate or result in the loss of pollen viability. The deficiency to metabolize sucrose to hexoses is caused by impairments of vacuolar and cell-wall bound invertases due to the down-regulation of the two genes, one encoding the vacuolar (*Ivr5*) and the other a cell-wall (*Ivr1*) isoform (Koonjul et al., 2004; Ba et al., 2019); and were found for wheat exposed to drought stress (Koonjul et al., 2004) or in cytoplasmic male sterile wheat plants (Ba et al., 2019). Therefore, we assume that invertases play important roles for accumulation of soluble sugars and, hence, wheat pollen germination.

Raffinose can serve as an important energy source for *in vitro* pollen germination. In wheat pollen, highest germination and longest pollen tube growth was stimulated on a medium containing raffinose as exclusive sugar. Raffinose is a trisaccharide composed of fructose, glucose, and galactose; and is cleaved into melibiose and fructose (Goetz and Roitsch, 1999). In wheat pollen, we found only minor amounts confirming the general knowledge that it is largely absent in cereals (McIntyre et al., 2012; Van den Ende, 2013). However, wheat invertases such as β-fructofuranosidase can hydrolyzed both, sucrose and raffinose (Goetz and Roitsch, 1999). Due to an amino acid modification, cell-wall bound plant invertases have even a higher substrate specificity and a higher relative cleavage rate for raffinose compared to sucrose (Goetz and Roitsch, 1999). The subsequent transport of the different cleavage products may appear actively and in parallel (Goetz et al., 2017). Fructose can be transported by POLYOL/MONOSACCHARIDE TRANSPORTER 1 (AtPMT1), 2 (AtPMT2) (Klepek et al., 2009), 5 (AtPMT5) proteins (Klepek et al., 2005), and a pollen-specific H+ -monosaccharide symporter STP6 (Scholz-Starke et al., 2003) across the pollen membrane. These transports are driven by a proton gradient (Slewinski, 2011; Rottmann et al., 2016). Their optimum is around pH 5.0 (AtPMT1 and AtPMT2) (Klepek et al., 2009) which may explain the higher efficiency in wheat pollen germination at pH 5.8 in comparison to 7.3. Summarizing, we assume that raffinose might be partly cleaved by cell-wall bound invertases and products are actively transported into wheat pollen to support pollen germination. Alternatively, the uptake of raffinose, fructose, and other cleavage products may occur simultaneously. However, the involvement of enzymes in sugar cleavage and uptake need to be explored further in wheat pollen in order to identify specific requirements for pollen germination.

Raffinose may support *in vitro* pollen germination in wheat by specific signaling and as a protecting agent. The substitution of raffinose or the supplement of maltose, sucrose, or sorbitol reduced dramatically wheat pollen germination (**Figure 3**). Also the raffinose components, such as fructose or galactose, failed to stimulate wheat pollen germination (Cheng and McComb, 1992). In Arabidopsis pollen, the hexokinase (HXK) enzyme, an important sensor for sugar metabolism and sugar signaling, has a lower sensitivity for fructose. Changes in the fructose to glucose ratio (Hirsche et al., 2017) or deficiencies in the glucose signaling contribute to normal pollen tube growth (Rottmann et al., 2018) indicating that changes in the sugar composition may have substantial effects on pollen germination and the signaling pathways.

Furthermore, raffinose is known as stress protective agent. In leaves, higher levels of galactinol and raffinose correlated with increased tolerance against drought, salinity, and cold stress and efficient protection against hydroxyl radicals (Taji et al., 2002; Nishizawa et al., 2008). It further provides protection to liposomes and is involved in the stabilization of cellular membranes under stress (Hincha et al., 2003). Nevertheless, pollen germination in the presence of a stigma was superior compared to *in vitro* pollen germination showing that the complex network of metabolic activities, signaling, and protection mechanism need to be further elucidate to improve the capability of pollen to germinate and grow on *in vitro* media.

### Pollen Germination Depends on Species, Line, and Environmental Conditions

The *in vitro* pollen germination is assumed to be partly genetically controlled. On the raffinose-based medium ‘Basic’, wheat pollen germination varied between 3% and 50% in 19 different lines and was often higher in spring-types compared to winter types. Also in rye, barley, and maize, pollen germinated between 3.3% and 42.4% (25 lines), between 0.4% and 13.6% (11 lines), and between 0.2% and 4.5% (4 lines), respectively. The composition of pollen wall, the exine, is presumably responsible, among other factors, for variations in *in vitro* germination and underlies various changes during pollen tube development. Therefore, a unique composition including the production of extraplastidial lipids and the storage of triacylglycerols in lipid droplets have evolved (Ischebeck, 2016). In wheat, the composition of extraplastidic phospholipids is dominated by 34:3 and 36:6 lipid molecular species which include linolenic acid (18:3) (Narayanan et al., 2018). The ratio between linoleic (18:2) and linolenic acid may vary as shown for oilseed rape pollen (Evans et al., 1990). Hoekstra et al. (1992) hypothesized that different degrees of poly-unsaturation may affect pollen viability and longevity. A higher degree of polyunsaturated fatty acids facilitates a greater membrane fluidity accelerating transport processes of cell wall precursors over the plasma membrane and, hence, stimulating fast pollen tube growth. Although lipids of minor quantities may also play important roles for pollen viability status (Jiang et al., 2015), the high degree of polyunsaturated fatty acids in wheat pollen might be prone to oxidative processes such as phenolic oxidation known for maize pollen (Žilić et al., 2014). Here, flavonoids, especially quercetin diglycoside, were found to protect pollen against oxidative stress. Pollen of sweet maize had the highest content of total phenolics and flavonoids, hence, the highest antioxidant capacity. Other protective compounds are cold, heat shock, and Fe-deficiency proteins (Jagadish et al., 2009). Therefore, we assume that lipid composition and variations in protective compounds may cause genotypic variations in pollen viability.

Environmental conditions and the time point of pollen shedding can cause strong variations in pollen germination. Here, the right anther stage needs to be identified to determine the optimum time point of pollen harvest and to test mature pollen. Thereby, anther dehiscence depends on the rupture of the septa, expansion of locule walls, pollen swelling prior to anthesis, and rupturing of the stomium (Liu et al., 2006) (**Supplementary Video S1**). In wheat, anthers located in the middle of a spike and at the outer florets of the spikelets tend to open and to shed mature pollen first. Afterwards, anthers located at the inner florets dehisce and the anthers of the basal and apical spikelets follow (Lukac et al., 2012). Pollen maturation and shedding can be triggered by moderate temperature increases. However, high temperature stress during anthesis affects pollen viability and reduces seed set (Jiang et al., 2015; Bheemanahalli et al., 2019). Pollen of maize is highly sensitive to desiccation; and pollen is non-viable in more than 300 m distance of the mother plants (Luna et al., 2001). The viability of cotton pollen can be reduced when flowers are directly exposed to sunlight (Burke et al., 2004). In contrast, the half-life can be extended under cloudy atmospheric conditions (Ge et al., 2011). Here, the carbohydrate concentration plays again an important role. Sucrose and starch increase in pollen due to the reduction of the metabolism under heat stress (Aloni et al., 2001). In the current study, constant greenhouse conditions were applied to plants used for pollen production. However, variations in pollen germination might be triggered by diverse environmental stresses occurring across the year. Therefore, pollen germination of the wheat line TRI 9102 may have ranged between 7.4 and 64.2%. In summary, optimal environmental conditions during plant production and mature pollen are required to gain high pollen quality and germination.

### Comparison of Viability Tests for Wheat Pollen

FDA staining, IF cytometry, *in vitro*, and stigmatic germination were able to distinguish fresh from stored wheat pollen. By comparing pollen viability of three lines (**Figure 5A**), results correlated highly between *in vitro* pollen germination and IF cytometry (r = 0.67, P < 0.001) and FDA staining (r = 0.54, P < 0.05). Higher pollen germination was found on stigmas for one line. At the stigma, nutritional supply and structural elements are assumed to be species specific and promote pollen germination. Fritz and Lukaszewski (1989) indicated that *in vivo* or semi *in vivo* assays were most accurate tests to assess pollen viability for wheat and triticale. However, difficulties in the identification of germinated pollen and high risks of incompatibilities (Dafni and Firmage, 2000) are unfavorable for quick reliable tests. A fast viability analysis is the measurement of dielectric properties of pollen by IF cytometry (Heidmann et al., 2016). In wheat, it can discriminate between fresh and stored pollen and has shown high correlations between results of IF cytometry and FDA staining for pollen of tomato, cucumber, and sweet pepper (Heidmann et al., 2016). Furthermore, also FDA staining has been proven to indicate pollen viability for cherry (La Porta and Roselli, 1991), potato (Trognitz, 1991), and acacia pollen (Sedgley and Harbard, 1993). Nevertheless, when the analysis of stored, non-viable pollen is absent such as shown for wheat (**Figure 6**), rye (**Supplementary Figure S3A**), barley (**Supplementary Figure S3B**), and maize (**Supplementary Figure S3C**), correlation coefficients between FDA staining and *in vitro* germination are rather low. We conclude that FDA staining and IF cytometry tend to overestimate the ability of pollen to germinate and produce long pollen tubes whereas the *in vitro* germination test requires specific modification for different species and perhaps lines. When optimal growth conditions for pollen are absent on medium, the *in vitro* pollen germination might give an underestimation of the ability of pollen to germinate.

Stored, non-viable wheat pollen was stained by the Alexander assay; hence, the test was excluded as viability test for pollen. The Alexander staining has been developed to identify the presence or absence of cytoplasm indicating sterility or maturity of pollen grains (Alexander, 1969). Therefore, Alexander staining is efficient when species produce a high number of aborted pollen (Atlagić et al., 2012; Silva et al., 2018). The use as viability test is disputed because stored and non-viable pollen having intact cytoplasm are stained and misinterpreted as viable (Heslop-Harrison et al., 1984). Comparable to the results in wheat, immature or incompletely formed pollen were stained in Johnson grass (*Sorghum halepense*) (Burke et al., 2007) and correlations between results of *in vitro* germination and Alexander staining were absent in asparagus pollen (Marcellán and Camadro, 1996). Therefore, we suggest that publications should clearly distinguish between germinability, stainability, and sterility/maturity if the Alexander staining is used.

## Conclusions

Wheat is known for short-lived, recalcitrant pollen which cannot be stored so far. To work toward an efficient storage approach, in the present study, we assessed the viability of fresh and stored wheat pollen using 157 different solid and liquid media and identified a solid medium based on raffinose as most appropriate. Although raffinose is not the main soluble sugar in pollen, wheat invertases have a higher affinity to cleave raffinose compared to sucrose which is highly abundant in antheres. Therefore, raffinose might be transported in parallel with its cleavage product through the exine. When raffinose is accumulated, it may function as a protective agent and stabilizes pollen membranes in addition. However, higher germination was achieved by stigmatic germination indicating complex structural and/or biochemical signals from the stigma that trigger higher percentages of pollen tube growth. *In vitro* germination of pollen was lower compared to pollen viability assessed by FDA and IF cytometry and varied between lines, species, and growth conditions. However, it is still elusive to which extend pollen classified as viable is able to germinate and grow. Therefore, it is recommendable to choose a combined approach of *in vitro* germination with FDA or IF cytometry in order to analyze the potential of pollen germination and viability correctly.

## Data Availability Statement

The datasets generated for this study are available on request to the corresponding author.

## Author Contributions

DI, AS, and MN designed the experiments. DI, JR, CK, and HR conducted the experiments. DI analyzed the data. AB provided substantial resources. DI and MN wrote the manuscript. All authors proofed and corrected the manuscript.

## Funding

This study was supported and publication fees provided by the Leibniz Institute of Plant Genetics and Crop Plant Research (IPK).

## Conflict of Interest

The authors declare that the research was conducted in the absence of any commercial or financial relationships that could be construed as a potential conflict of interest.

## Abbreviations

FDA, fluorescein diacetate; IF, impedance flow cytometry; IAA, indole acetic acid; GA_3_, gibberellic acid; PEG, polyethylene glycol; Pep, peptone water; EACA, ε-aminocaproic acid.
