# Supplementary material for: Assessment of Pollen Viability for Wheat
Source: Front Plant Sci. 2020 Jan 22;10:1588. doi: 10.3389/fpls.2019.01588 (PMC6987437; doi:10.3389/fpls.2019.01588)
Supplement: Supplementary file 1 [file DataSheet_1.docx]

Supplementary Material

Assessment of pollen viability for wheat

Daniela Impe^1^, Janka Reiz^1^, Claudia Köpnick^1^, Hardy Rolletschek^2^, Andreas Börner^1^, Angelika Senula^1^, Manuela Nagel^1^*

# Supplementary Tables

Supplementary Table S1 Details of the tested genotypes. Wheat, rye and barley accessions were obtained from the Federal *Ex situ* Gene Bank for Agricultural and Horticultural Plants in Gatersleben, Germany (https://doi.org/10.5447/ipk/2019/9). Maize genotypes were obtained from the Maize Genetics Stock Center, USA (http://maizecoop.cropsci.uiuc.edu).

| **Accession name** | **Accession number** | **Annuity** | **Biotype** | **Botanical name** | **Country of donation/approval** | | **Donor/  breeding company** | | **Year of access/ approval** | |
| --- | --- | --- | --- | --- | --- | --- | --- | --- | --- | --- |
| Ferrum |  | winter | variety | *Triticum aestivum* L. | Germany | | KWS Lochow GmbH | | 2012 | |
| Hermann |  | winter | variety | *Triticum aestivum* L. | Germany | | LIMAGRAIN GmbH | | 2004 | |
| Piko |  | winter | variety | *Triticum aestivum* L. | Germany | | NORDSAAT Saatzucht GmbH | | 1994 | |
| Odyssee |  | winter | variety | *Triticum aestivum* L. | United Kingdom | | Syngenta International AG | | 2011 | |
| Dialog |  | winter | variety | *Triticum aestivum* L. | France | | KWS UK Ltd. | | 2008 | |
| Einstein |  | winter | variety | *Triticum aestivum* L. | United Kingdom | | LIMAGRAIN GmbH | | 2001 | |
| Henrik |  | winter | variety | *Triticum aestivum* L. | Belgium | | LIMAGRAIN GmbH | | 2009 | |
| Genius |  | winter | variety | *Triticum aestivum* L. | Germany | | NORDSAAT Saatzucht GmbH | | 2010 | |
| Mulan |  | winter | variety | *Triticum aestivum* L. | Germany | | NORDSAAT Saatzucht GmbH | | 2006 | |
| Bussard |  | winter | variety | *Triticum aestivum* L. var. *lutescens* (Alef.) Mansf. | Germany | | KWS Lochow GmbH | | 1990 | |
| Milaneco |  | winter | variety | *Triticum aestivum* L. | Germany | | KWS Lochow GmbH | | 2013 | |
| Lang-Dörflers Braunweizen Walthari | TRI 1340 | winter | landrace | *Triticum aestivum* L. var. *milturum* (Alef.) Mansf. | Germany | | Lang-Doerfler Niedertraubling/Ndb. | | 1947 | |
| Bezostaja 1 | TRI 6747 | winter | landrace | *Triticum aestivum* L. var. lutescens (Alef.) Mansf. | Soviet Union | | N.I. Vavilov All-Russian Scientific Research Institute of Plant Genetic Resources | | 1963 | |
| Rode Ris | TRI 8280 | winter | landrace | *Triticum aestivum* L. var. *lutescens* (Alef.) Mansf. | Netherland | | Institute of Horticultural Plant Breeding Wageningen | | 1967 | |
| Triso |  | spring | variety | *Triticum aestivum* L. | Germany | | Deutsche Saatveredelung AG | | 1996 | |
| Melissos | TRI 29940 | spring | variety | *Triticum aestivum* L. | Germany | | Fa. Strube Saatzucht KG | | 2003 | |
| Melon | TRI 29513 | spring | variety | *Triticum aestivum* L. var. *lutescens* (Alef.) Mansf. | Germany | | Fa. Strube Saatzucht KG | | 1997 | |
| Kalistos | TRI 29475 | spring | variety | *Triticum aestivum* L. var. *lutescens* (Alef.) Mansf. | Germany | | Fa. Strube Saatzucht KG | | 1999 | |
| Combi | TRI 29911 | spring | variety | *Triticum aestivum* L. | Germany | | Saatzucht Engelen Büchling e.K. | | 1990 | |
|  | TRI 4399 | spring | landrace | *Triticum aestivum* L. var. *icterinum* (Alef.) Mansf. | Europe | | Agricultural Botanical Garden Szentes, Hungary | | 1956 | |
|  | TRI 9102 | spring | landrace | *Triticum aestivum* L. var. *ferrugineum* (Alef.) Mansf. | Austria | | Mayr, alpine landrace collection | | 1922 | |
|  | TRI 2679 | spring | landrace | *Triticum aestivum* L. var. *aestivum* | India | | Herrlich, Northwest-India-Nepal | | 1937 | |
|  | TRI 2443 | spring | landrace | *Triticum aestivum* L. var. *aestivum* | Nepal | | Herrlich, Northwest-India-Nepal | | 1937 | |
|  | TRI 8392 | spring | landrace | *Triticum aestivum* L. var. *aestivum* | Mongolia | | Mongolia collection mission | | 1964 | |
|  | TRI 8891 | spring | landrace | *Triticum aestivum* L. var. *aestivum* | Austria | | Mayr, alpine landrace collection | | 1922 | |
|  | TRI 13752 | spring | landrace | *Triticum aestivum* L. var. *aestivum* | Lybia | | Lybia collection mission | | 1982 | |
|  | R 864 | spring | landrace | *Secale cereale* L. subsp. *cereale* | Georgia | | Georgia collection mission | | 1982 | |
|  | R 687 | spring | landrace | *Secale cereale* L. subsp. *cereale* | Poland | | Poland collection mission | | 1976 | |
| Tiroler Sommerroggen | R 979 | spring | landrace | *Secale cereale* L. subsp. *cereale* | Austria | | Austria collection mission | | 1986 | |
|  | R 594 | spring | landrace | *Secale cereale* L. subsp. *cereale* | Czech Republik | | Kühn, Brno | | 1975 | |
|  | R 2034 | spring | landrace | *Secale cereale* L. subsp. *cereale* | Russia | | N.I. Vavilov All-Russian Scientific Research Institute of Plant Genetic Resources | | 2015 | |
| SOMRO | R 2480 | spring | variety | *Secale cereale* L. subsp. *cereale* | Germany | | Lochow-Petkus GmbH | | 1977 | |
| Polko | R 257 | spring | variety | *Secale cereale* L. subsp. *cereale* | South Africa | | Institute of Tropical and Subtropical Agriculture, Leipzig | | 1966 | |
| Beka | R 1470 | spring | variety | *Secale cereale* L. subsp. *cereale* | Poland | | IPK Genebank Gülzow | | 1967 | |
| Petkuser Sommerroggen | R 2319 | spring | variety | *Secale cereale* L. subsp. *cereale* | Germany | | IHAR Radzikow | | 1997 | |
| Sorom | R 2263 | spring | variety | *Secale cereale* L. subsp. *cereale* | Germany | | IPK Genbank Gülzow | | 1997 | |
|  | R 1185 | winter | landrace | *Secale cereale* L. subsp. *cereale* | Albania | | Albania collection mission | | 1994 | |
| Anatolien | R 1874 | winter | landrace | *Secale cereale* L. subsp. *cereale* | Turkey | | IPK Genebank Gülzow | | 1997 | |
|  | R 674 | winter | landrace | *Secale cereale* L. subsp. *cereale* | Poland | | Poland collection mission | | 1976 | |
|  | R 708 | winter | landrace | *Secale cereale* L. subsp. *cereale* | Slowakia | | CSSR collection mission | | 1977 | |
|  | R 973 | winter | landrace | *Secale cereale* L. subsp. *cereale* | Georgia | | Georgia collection mission | | 1986 | |
| Plato | R 2938 | winter | variety | *Secale cereale* L. subsp. *cereale* | Germany | | Hybro Saatzucht GmbH | | 2001 | |
| Warko | R 2937 | winter | variety | *Secale cereale* L. subsp. *cereale* | Poland | | DNKO Hodowla Roslin Sp. zo.o. | | 2000 | |
| Hacada | R 2936 | winter | variety | *Secale cereale* L. subsp. *cereale* | Germany | | KWS Lochow GmbH | | 1993 | |
| Motto | R 2935 | winter | variety | *Secale cereale* L. subsp. *cereale* | Poland | | DNKO Hodowla Roslin Sp. zo.o. | | 1991 | |
| Halo | R 2934 | winter | variety | *Secale cereale* L. subsp. *cereale* | Germany | | KWS Lochow GmbH | | 1977 | |
| Boruus |  | winter | variety | *Secale cereale* L. subsp. *cereale* |  | |  | |  | |
| Turbo |  | winter | variety | *Secale cereale* L. subsp. *cereale* | Poland | | IPK Genebank Gülzow | | 2000 | |
| Borfuro |  | winter | variety | *Secale cereale* L. subsp. *cereale* | Germany | | Saatzucht Steinach GmbH | | 1996 | |
| Visello |  | winter | variety | *Secale cereale* L. subsp. *cereale* | Germany | | KWS Lochow GmbH | | 2006 | |
| Boresto |  | winter | variety | *Secale cereale* L. subsp. *cereale* | Germany | | Saatzucht Steinach GmbH | | 2000 | |
|  | HOR 4710 | spring | landrace | *Hordeum vulgare* L. convar. *distichon* (L.) Alef. var. *nutans* (Rode) Alef. | Germany | | VEG Saatzucht Boldebuck | | 1972 | |
|  | HOR 4706 | spring | landrace | *Hordeum vulgare* L. convar. *distichon* (L.) Alef. var. *nutans* (Rode) Alef. | Germany | | VEG Saatzucht Boldebuck | | 1972 | |
|  | HOR 2347 | spring | landrace | *Hordeum vulgare* L. convar. *distichon* (L.) Alef. var. *erectum* (Rode) Alef | Germany | | Schiemann Germany | | 1990 | |
|  | HOR 2901 | spring | landrace | *Hordeum vulgare* L. convar. *vulgare* var. *hybernum* Viborg | El Salvador | | Schwatzi, Central America collection mission | | 1958 | |
| Belana |  | spring | variety | *Hordeum vulgare* L. convar. *distichon* (L.) Alef. var. *nutans* (Rode) Alef. | Germany | | NORDSAAT Saatzucht GmbH | | 2003 | |
| Orthega |  | spring | variety | *Hordeum vulgare* L. convar. *distichon* (L.) Alef. var. *nutans* (Rode) Alef. | Germany | | KWS Lochow GmbH | | 1996 | |
|  | HOR 6974 | spring | variety | *Hordeum vulgare* L. convar. *distichon* (L.) Alef. var. *nutans* (Rode) Alef. | Germany | | VEG Saatzucht Derenburg | | 1974 | |
| Minerva | HOR 2492 | spring | variety | *Hordeum vulgare* L. convar. *distichon* (L.) Alef. var. *nutans* (Rode) Alef. | The Netherlands | | University of Agriculture, Wageningen | | 1990 | |
| Saale | HOR 2441 | spring | variety | *Hordeum vulgare* L. convar. *distichon* (L.) Alef. var. *nutans* (Rode) Alef. | Germany | | Institute for Plant Breeding, Halle | | 1990 | |
| Berendstets | HOR 3021 | spring | variety | *Hordeum vulgare* L. convar. *vulgare* var. *hybernum* Viborg | Germany | | Biologische Bundesanstalt Braunschweig | | 2000 | |
| Hiland | HOR 2754 | spring | variety | *Hordeum vulgare* L. convar. *vulgare* var. *rikotense* Regel | USA | | Barley collection, Beltsville | | 2000 | |
| PH 207 |  | spring | Inbred line | *Zea mays* L. | USA |  | |  | |  |
| A188 |  | spring | Inbred line | *Zea mays* L. | USA |  | |  | |  |
| B73 |  | spring | Inbred line | *Zea mays* L. | USA |  | |  | |  |
| A183 |  | spring | Inbred line | *Zea mays* L. | USA |  | |  | |  |

Supplementary Table S2 Pollen tube growth, length and frequency of pollen bursting on liquid growth media. Liquid media are based on protocols following Cheng and McComb (1992) (Cheng), Jayaprakash, et al. (2015) (Jaya), Jian, et al. (2014) (Jian) and were modified in micro- and macronutrients and pH. Frequency of pollen tubes was estimated and assigned as low, medium and high when one, two to ten or more than ten pollen, respectively, developed pollen tubes. Pollen tube length was characterized based on the pollen diameter and characterized as short (smaller than the pollen diameter), medium (between the one- and twofold diameter) and long (more than the twofold diameter) (Supplementary Figure S1). Frequency of pollen bursting refers to a single pollen (low), half of the pollen batch (medium) or the complete pollen batch which bursted. PEG, polyethylene glycol.

| **Protocols** | **Sucrose [mM]** | **Maltose [mM]** | **Raffinose [mM]** | **pH** | **H_3_BO_3_ [mM]** | **CaCl_2_·2H_2_O [mM]** | **Ca(NO_3_)_2_·2H_2_O [mM]** | **MgS [mM]** | **KNO_3_ [mM]** | **PEG 4000 [g L^-1^]** | **Frequency of pollen tubes** | **Pollen tube length** | **Frequency of pollen bursting** |
| --- | --- | --- | --- | --- | --- | --- | --- | --- | --- | --- | --- | --- | --- |
| Cheng | 300 | - | - | 5.8 | 1.62 | 2.04 | - | - | - | - | - | - | - |
| Cheng | 300 | - | - | 7.3 | 1.62 | 2.04 | - | - | - | - | - | - | - |
| Cheng | 600 | - | - | 5.8 | 1.62 | 2.04 | - | - | - | - | - | - | - |
| Cheng | 600 | - | - | 7.3 | 1.62 | 2.04 | - | - | - | - | - | - | - |
| Cheng | 750 | - | - | 5.8 | 1.62 | 2.04 | - | - | - | - | - | - | - |
| Cheng | 750 | - | - | 7.3 | 1.62 | 2.04 | - | - | - | - | - | - | - |
| Cheng | 900 | - | - | 5.8 | 1.62 | 2.04 | - | - | - | - | - | - | - |
| Cheng | 900 | - | - | 7.3 | 1.62 | 2.04 | - | - | - | - | - | - | - |
| Cheng | - | 300 | - | 5.8 | 1.62 | 2.04 | - | - | - | - | - | - | - |
| Cheng | - | 300 | - | 7.3 | 1.62 | 2.04 | - | - | - | - | - | - | - |
| Cheng | - | 600 | - | 5.8 | 1.62 | 2.04 | - | - | - | - | - | - | - |
| Cheng | - | 600 | - | 7.3 | 1.62 | 2.04 | - | - | - | - | - | - | - |
| Cheng | - | 750 | - | 5.8 | 1.62 | 2.04 | - | - | - | - | - | - | - |
| Cheng | - | 750 | - | 7.3 | 1.62 | 2.04 | - | - | - | - | - | - | - |
| Cheng | - | 900 | - | 5.8 | 1.62 | 2.04 | - | - | - | - | - | - | - |
| Cheng | - | 900 | - | 7.3 | 1.62 | 2.04 | - | - | - | - | - | - | - |
| Cheng | - | - | 300 | 5.8 | 1.62 | 2.04 | - | - | - | - | low | - | low |
| Cheng | - | - | 300 | 7.3 | 1.62 | 2.04 | - | - | - | - | - | - | - |
| Cheng | - | - | 600 | 5.8 | 1.62 | 2.04 | - | - | - | - | low | medium | medium |
| Cheng | - | - | 600 | 7.3 | 1.62 | 2.04 | - | - | - | - | high | long | low |
| Cheng | - | - | 750 | 5.8 | 1.62 | 2.04 | - | - | - | - | low | short | low |
| Cheng | - | - | 750 | 7.3 | 1.62 | 2.04 | - | - | - | - | medium | medium | medium |
| Cheng | 300 | - | - | 5.8 | 0.81 | 2.04 | - | - | - | - | - | - | - |
| Cheng | 300 | - | - | 7.3 | 0.81 | 2.04 | - | - | - | - | - | - | - |
| Cheng | 600 | - | - | 5.8 | 0.81 | 2.04 | - | - | - | - | - | - | - |
| Cheng | 600 | - | - | 7.3 | 0.81 | 2.04 | - | - | - | - | - | - | - |
| Cheng | 750 | - | - | 5.8 | 0.81 | 2.04 | - | - | - | - | low | - | low |
| Cheng | 750 | - | - | 7.3 | 0.81 | 2.04 | - | - | - | - | - | - | - |
| Cheng | 900 | - | - | 5.8 | 0.81 | 2.04 | - | - | - | - | - | - | - |
| Cheng | 900 | - | - | 7.3 | 0.81 | 2.04 | - | - | - | - | - | - | - |
| Cheng | - | 300 | - | 5.8 | 0.81 | 2.04 | - | - | - | - | - | - | - |
| Cheng | - | 300 | - | 7.3 | 0.81 | 2.04 | - | - | - | - | - | - | - |
| Cheng | - | 600 | - | 5.8 | 0.81 | 2.04 | - | - | - | - | - | - | - |
| Cheng | - | 600 | - | 7.3 | 0.81 | 2.04 | - | - | - | - | - | - | - |
| Cheng | - | 750 | - | 5.8 | 0.81 | 2.04 | - | - | - | - | - | - | - |
| Cheng | - | 750 | - | 7.3 | 0.81 | 2.04 | - | - | - | - | - | - | - |
| Cheng | - | 900 | - | 5.8 | 0.81 | 2.04 | - | - | - | - | - | - | - |
| Cheng | - | 900 | - | 7.3 | 0.81 | 2.04 | - | - | - | - | - | - | - |
| Cheng | - | - | 300 | 5.8 | 0.81 | 2.04 | - | - | - | - | - | - | - |
| Cheng | - | - | 300 | 7.3 | 0.81 | 2.04 | - | - | - | - | - | - | - |
| Cheng | - | - | 600 | 5.8 | 0.81 | 2.04 | - | - | - | - | low | - | low |
| Cheng | - | - | 600 | 7.3 | 0.81 | 2.04 | - | - | - | - | high | short | low |
| Cheng | - | - | 750 | 5.8 | 0.81 | 2.04 | - | - | - | - | low | - | medium |
| Cheng | - | - | 750 | 7.3 | 0.81 | 2.04 | - | - | - | - | high | medium | low |
| Cheng | 300 | - | - | 5.8 | 1.62 | 1.02 | - | - | - | - | - | - | - |
| Cheng | 300 | - | - | 7.3 | 1.62 | 1.02 | - | - | - | - | - | - | - |
| Cheng | 600 | - | - | 5.8 | 1.62 | 1.02 | - | - | - | - | - | - | - |
| Cheng | 600 | - | - | 7.3 | 1.62 | 1.02 | - | - | - | - | - | - | - |
| Cheng | 750 | - | - | 5.8 | 1.62 | 1.02 | - | - | - | - | - | - | - |
| Cheng | 750 | - | - | 7.3 | 1.62 | 1.02 | - | - | - | - | - | - | - |
| Cheng | 900 | - | - | 5.8 | 1.62 | 1.02 | - | - | - | - | - | - | - |
| Cheng | 900 | - | - | 7.3 | 1.62 | 1.02 | - | - | - | - | - | - | - |
| Cheng | - | 300 | - | 5.8 | 1.62 | 1.02 | - | - | - | - | - | - | - |
| Cheng | - | 300 | - | 7.3 | 1.62 | 1.02 | - | - | - | - | - | - | - |
| Cheng | - | 600 | - | 5.8 | 1.62 | 1.02 | - | - | - | - | - | - | - |
| Cheng | - | 600 | - | 7.3 | 1.62 | 1.02 | - | - | - | - | - | - | - |
| Cheng | - | 750 | - | 5.8 | 1.62 | 1.02 | - | - | - | - | - | - | - |
| Cheng | - | 750 | - | 7.3 | 1.62 | 1.02 | - | - | - | - | - | - | - |
| Cheng | - | 900 | - | 5.8 | 1.62 | 1.02 | - | - | - | - | - | - | - |
| Cheng | - | 900 | - | 7.3 | 1.62 | 1.02 | - | - | - | - | - | - | - |
| Cheng | - | - | 300 | 5.8 | 1.62 | 1.02 | - | - | - | - | low | - | low |
| Cheng | - | - | 300 | 7.3 | 1.62 | 1.02 | - | - | - | - | low | short | medium |
| Cheng | - | - | 600 | 5.8 | 1.62 | 1.02 | - | - | - | - | high | short | medium |
| Cheng | - | - | 600 | 7.3 | 1.62 | 1.02 | - | - | - | - | high | medium | low |
| Cheng | - | - | 750 | 5.8 | 1.62 | 1.02 | - | - | - | - | high | short | low |
| Cheng | - | - | 750 | 7.3 | 1.62 | 1.02 | - | - | - | - | high | short | low |
| Jaya | 300 | - | - | 5.8 | 0.81 | - | 0.13 | 0.81 | 0.99 | 130 | - | - | - |
| Jaya | 300 | - | - | 7.3 | 0.81 | - | 0.13 | 0.81 | 0.99 | 130 | - | - | - |
| Jaya | 600 | - | - | 5.8 | 0.81 | - | 0.13 | 0.81 | 0.99 | 130 | - | - | - |
| Jaya | 600 | - | - | 7.3 | 0.81 | - | 0.13 | 0.81 | 0.99 | 130 | - | - | - |
| Jaya | 750 | - | - | 5.8 | 0.81 | - | 0.13 | 0.81 | 0.99 | 130 | - | - | - |
| Jaya | 750 | - | - | 7.3 | 0.81 | - | 0.13 | 0.81 | 0.99 | 130 | - | - | - |
| Jaya | 900 | - | - | 5.8 | 0.81 | - | 0.13 | 0.81 | 0.99 | 130 | - | - | - |
| Jaya | 900 | - | - | 7.3 | 0.81 | - | 0.13 | 0.81 | 0.99 | 130 | - | - | - |
| Jaya | - | 300 | - | 5.8 | 0.81 | - | 0.13 | 0.81 | 0.99 | 130 | low | long | medium |
| Jaya | - | 300 | - | 7.3 | 0.81 | - | 0.13 | 0.81 | 0.99 | 130 | - | - | - |
| Jaya | - | 600 | - | 5.8 | 0.81 | - | 0.13 | 0.81 | 0.99 | 130 | - | - | - |
| Jaya | - | 600 | - | 7.3 | 0.81 | - | 0.13 | 0.81 | 0.99 | 130 | - | - | - |
| Jaya | - | 750 | - | 5.8 | 0.81 | - | 0.13 | 0.81 | 0.99 | 130 | low | - | low |
| Jaya | - | 750 | - | 7.3 | 0.81 | - | 0.13 | 0.81 | 0.99 | 130 | - | - | - |
| Jaya | - | 900 | - | 5.8 | 0.81 | - | 0.13 | 0.81 | 0.99 | 130 | - | - | - |
| Jaya | - | 900 | - | 7.3 | 0.81 | - | 0.13 | 0.81 | 0.99 | 130 | - | - | - |
| Jaya | - | - | 300 | 5.8 | 0.81 | - | 0.13 | 0.81 | 0.99 | 130 | low | - | low |
| Jaya | - | - | 300 | 7.3 | 0.81 | - | 0.13 | 0.81 | 0.99 | 130 | medium | short | low |
| Jaya | - | - | 600 | 5.8 | 0.81 | - | 0.13 | 0.81 | 0.99 | 130 | - | - | - |
| Jaya | - | - | 600 | 7.3 | 0.81 | - | 0.13 | 0.81 | 0.99 | 130 | - | - | - |
| Jaya | - | - | 750 | 5.8 | 0.81 | - | 0.13 | 0.81 | 0.99 | 130 | - | - | - |
| Jaya | - | - | 750 | 7.3 | 0.81 | - | 0.13 | 0.81 | 0.99 | 130 | - | - | - |
| Jian | 300 | - | - | 5.8 | 0.65 | - | 2.96 | 0.81 | 0.99 | 100 | low | - | low |
| Jian | 300 | - | - | 7.3 | 0.65 | - | 2.96 | 0.81 | 0.99 | 100 | - | - | - |
| Jian | 600 | - | - | 5.8 | 0.65 | - | 2.96 | 0.81 | 0.99 | 100 | - | - | - |
| Jian | 600 | - | - | 7.3 | 0.65 | - | 2.96 | 0.81 | 0.99 | 100 | - | - | - |
| Jian | 750 | - | - | 5.8 | 0.65 | - | 2.96 | 0.81 | 0.99 | 100 | - | - | - |
| Jian | 750 | - | - | 7.3 | 0.65 | - | 2.96 | 0.81 | 0.99 | 100 | - | - | - |
| Jian | 900 | - | - | 5.8 | 0.65 | - | 2.96 | 0.81 | 0.99 | 100 | - | - | - |
| Jian | 900 | - | - | 7.3 | 0.65 | - | 2.96 | 0.81 | 0.99 | 100 | - | - | - |
| Jian | - | 300 | - | 5.8 | 0.65 | - | 2.96 | 0.81 | 0.99 | 100 | low | - | medium |
| Jian | - | 300 | - | 7.3 | 0.65 | - | 2.96 | 0.81 | 0.99 | 100 | - | - | - |
| Jian | - | 600 | - | 5.8 | 0.65 | - | 2.96 | 0.81 | 0.99 | 100 | - | - | - |
| Jian | - | 600 | - | 7.3 | 0.65 | - | 2.96 | 0.81 | 0.99 | 100 | - | - | - |
| Jian | - | 750 | - | 5.8 | 0.65 | - | 2.96 | 0.81 | 0.99 | 100 | - | - | - |
| Jian | - | 750 | - | 7.3 | 0.65 | - | 2.96 | 0.81 | 0.99 | 100 | - | - | - |
| Jian | - | 900 | - | 5.8 | 0.65 | - | 2.96 | 0.81 | 0.99 | 100 | - | - | - |
| Jian | - | 900 | - | 7.3 | 0.65 | - | 2.96 | 0.81 | 0.99 | 100 | - | - | - |
| Jian | - | - | 300 | 5.8 | 0.65 | - | 2.96 | 0.81 | 0.99 | 100 | - | - | - |
| Jian | - | - | 300 | 7.3 | 0.65 | - | 2.96 | 0.81 | 0.99 | 100 | high | short | low |
| Jian | - | - | 600 | 5.8 | 0.65 | - | 2.96 | 0.81 | 0.99 | 100 | low | - | low |
| Jian | - | - | 600 | 7.3 | 0.65 | - | 2.96 | 0.81 | 0.99 | 100 | - | - | - |
| Jian | - | - | 750 | 5.8 | 0.65 | - | 2.96 | 0.81 | 0.99 | 100 | - | - | - |
| Jian | - | - | 750 | 7.3 | 0.65 | - | 2.96 | 0.81 | 0.99 | 100 | medium | short | low |

**Supplementary Table S3 Pollen germination and frequency of pollen bursting on solid media.** Liquid media stimulating pollen tube growth were solidified and modified in micro- and macronutrients and pH. Mean and standard deviations of germinated and bursted pollen counted of the genotypes ‘Ferrum’, ‘Piko’, ‘Hermann’are given in percentage. Cheng, Cheng and McOmb (1992); Jaya, Jayaprakash, et al. (2015); PEG, polyethylene glycol; EACA, ε-aminocaproic acid

| **Protocols** | **Sucrose [mM]** | **Maltose [mM]** | **Raffinose [mM]** | **pH** | **H_3_BO_3_ [mM]** | **CaCl_2_·2H_2_O [mM]** | **Ca(NO_3_)_2_·2H_2_O [mM]** | **EACA [mM]** | **Peptone water [mg L^-1^]** | **PEG 4000 [g L^-1^]** | **PEG 8000 [g L^-1^]** | **Pollen germination [%]** | **Pollen bursting [%]** |
| --- | --- | --- | --- | --- | --- | --- | --- | --- | --- | --- | --- | --- | --- |
| Jaya | - | 584 | - | 5.8 | 0.81 | - | **0.13** | **3.81** | **100** | **130** | - | - | - |
| Jaya | - | 584 | - | 5.8 | 0.81 | - | **0.13** | **3.81** | **-** | **130** | - | - | - |
| Jaya | - | 584 | - | 5.8 | 0.81 | - | **0.13** | **-** | **100** | **130** | - | - | - |
| Jaya | - | 584 | - | 5.8 | 0.81 | - | **0.13** | **3.81** | **100** | **-** | - | - | - |
| Jaya | - | 584 | - | 5.8 | 0.81 | **1.02** | **-** | **3.81** | **100** | **130** | - | - | - |
| Jaya | - | 584 | - | 5.8 | **1.62** | - | 0.13 | 3.81 | 100 | 130 | - | - | - |
| Jaya | - | 584 | - | 5.8 | **2.43** | - | 0.13 | 3.81 | 100 | 130 | - | - | - |
| Jaya | - | 584 | - | 5.8 | **4.85** | - | 0.13 | 3.81 | 100 | 130 | - | - | - |
| Jaya | - | 584 | - | 5.8 | 0.81 | - | **0.42** | 3.81 | 100 | 130 | - | - | - |
| Jaya | - | 584 | - | 5.8 | 0.81 | - | **0.64** | 3.81 | 100 | 130 | - | - | - |
| Jaya | - | 584 | - | 5.8 | 0.81 | - | **2.12** | 3.81 | 100 | 130 | - | - | - |
| Jaya | - | 584 | - | 5.8 | 0.81 | - | **4.23** | 3.81 | 100 | 130 | - | - | - |
| Jaya | - | 584 | - | 5.8 | 0.81 | - | 0.13 | 3.81 | 100 | **50** | - | - | - |
| Jaya | - | 584 | - | 5.8 | 0.81 | - | 0.13 | 3.81 | 100 | **100** | - | - | - |
| Jaya | - | 584 | - | 5.8 | 0.81 | - | 0.13 | 3.81 | 100 | - | **50** | - | - |
| Jaya | - | 584 | - | 5.8 | 0.81 | - | 0.13 | 3.81 | 100 | - | **100** | - | - |
| Jaya | - | **876** | - | 5.8 | 0.81 | - | 0.13 | 3.81 | 100 | 130 | - | - | - |
| Jaya | - | **1460** | - | 5.8 | 0.81 | - | 0.13 | 3.81 | 100 | 130 | - | - | - |
| Jaya | **584** | - | - | 5.8 | 0.81 | - | 0.13 | 3.81 | 100 | 130 | - | - | - |
| Jaya | **876** | - | - | 5.8 | 0.81 | - | 0.13 | 3.81 | 100 | 130 | - | - | - |
| Jaya | **1460** | - | - | 5.8 | 0.81 | - | 0.13 | 3.81 | 100 | 130 | - | - | - |
| Jaya | - | - | **198** | 5.8 | 0.81 | - | 0.13 | 3.81 | 100 | 130 | - | - | - |
| Jaya | - | - | **396** | 5.8 | 0.81 | - | 0.13 | 3.81 | 100 | 130 | - | - | - |
| Jaya | - | - | **505** | 5.8 | 0.81 | - | 0.13 | 3.81 | 100 | 130 | - | - | - |
| Cheng | - | - | 252 | **5.8** | **0.81** | **2.04** | - | - | - | - | - | - | - |
| Cheng | - | - | 252 | **7.3** | **0.81** | **2.04** | - | - | - | - | - | - | - |
| Cheng | - | - | 252 | **5.8** | **1.62** | **1.02** | - | - | - | - | - | - | - |
| Cheng | - | - | 252 | **7.3** | **1.62** | **1.02** | - | - | - | - | - | - | - |
| Cheng | **731** | **-** | **-** | 5.8 | **0.81** | **2.04** | - | 3.81 | 100 | - | - | - | - |
| Cheng | **-** | **833** | **-** | 5.8 | **0.81** | **2.04** | - | 3.81 | 100 | - | - | - | - |
| Cheng | **-** | **-** | **594** | 5.8 | **0.81** | **2.04** | - | 3.81 | 100 | - | - | 32.7±14.2 | 61.7±18.3 |
| Cheng | **731** | **-** | **-** | 5.8 | **1.62** | **1.02** | - | 3.81 | 100 | - | - | - | - |
| Cheng | **-** | **833** | **-** | 5.8 | **1.62** | **1.02** | - | 3.81 | 100 | - | - | - | - |
| Cheng | **-** | **-** | **594** | 5.8 | **1.62** | **1.02** | - | 3.81 | 100 | - | - | 13.3±10.4 | 65.8±19.8 |

# Supplementary Figures


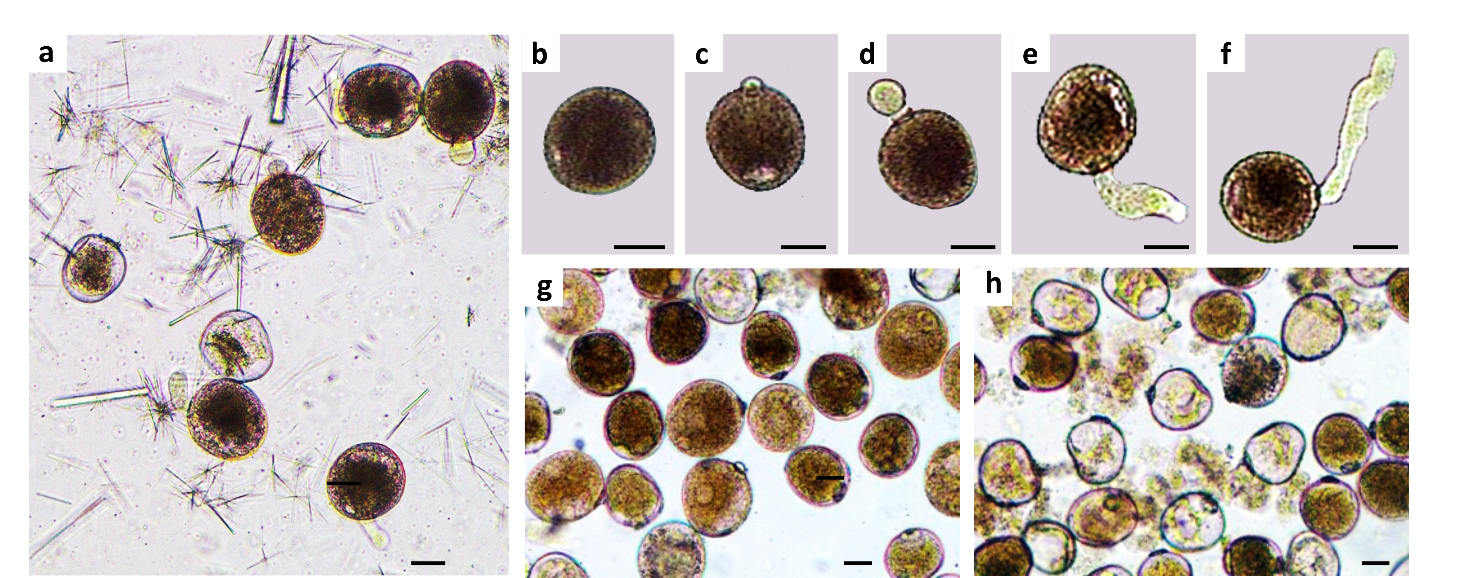


**Supplementary Figure S1 Pollen tube development and assessment in liquid media.** a) Raffinose, the main sugar in liquid media, crystallizes rapidly at room temperature. Therefore, exact evaluation was hampered and, pollen was categorized into b and c) absent pollen tubes; d) short pollen tubes (smaller than the pollen diameter); e) medium pollen tubes (between the one- and twofold diameter); and f) long pollen tubes (more than the twofold diameter) (Supplementary Table S2). g and h) Frequency of pollen bursting was dependent on the genotype and ranged between low (g) and high (h). Scale bar = 30 µm.


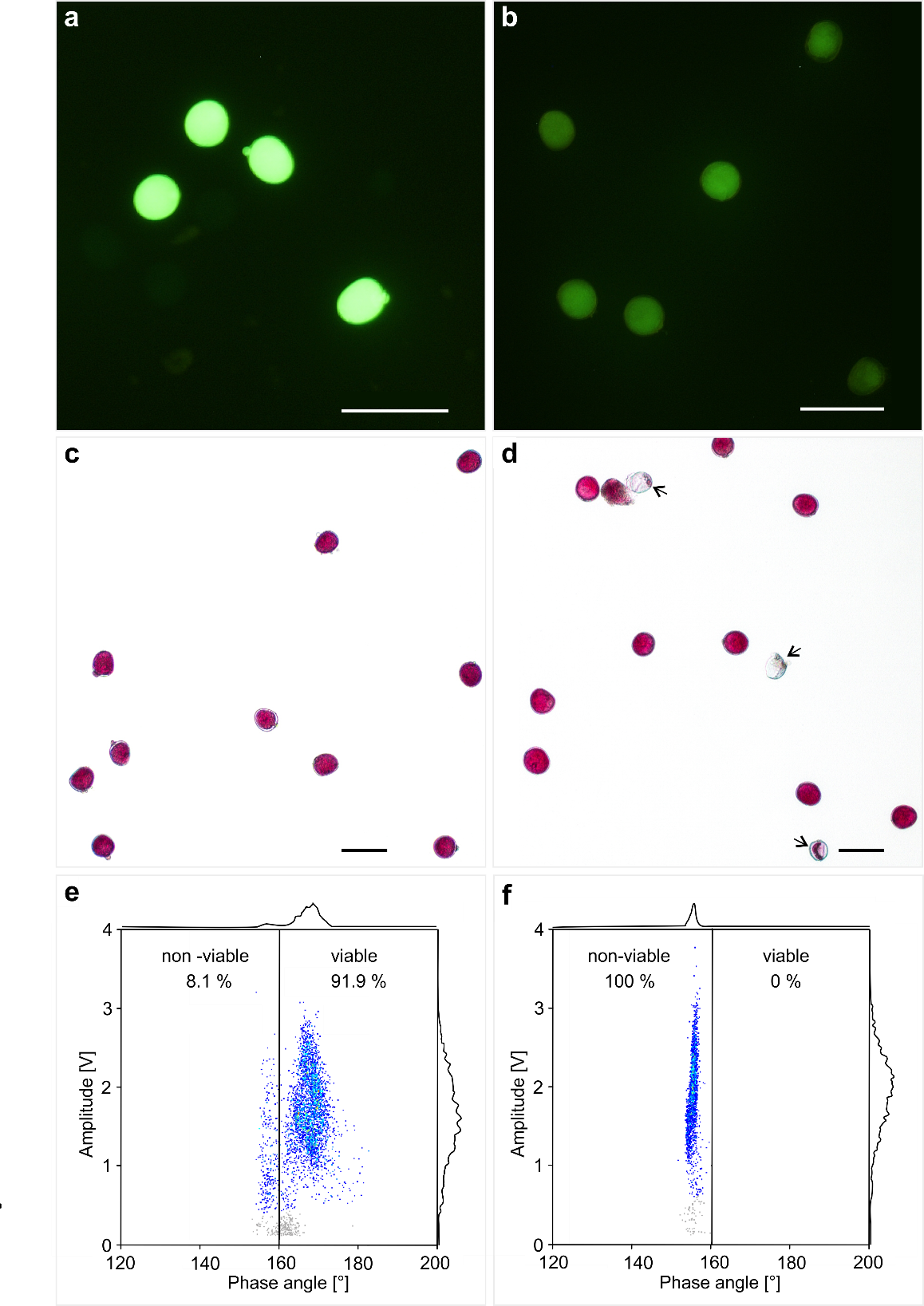


Supplementary Figure S2. Results of different pollen viability assays. a) Fluorescein diacetate (FDA) show different results for fresh and b) stored pollen assessed at 95.0 % and 0.1 % pollen viability. Fresh pollen is shown in a bright green whereas stored pollen has a uniform background fluorescence. c) Red pollen stained by Alexander solution indicate pollen viability whereas d) the absence of staining show sterile pollen (arrows). The Alexander stain cannot distinguish between fresh and stored pollen. Therefore, pollen of 0 % *in vitro* germination are also colored in red. Scale bar = 100 µm. e) Histograms and dot plots are shown after impedance flow cytometry using the Ampha Z32 (Amphasys, Lucerne, Switzerland) at 1 MHz. Number of pollen were counted and viability of fresh pollen samples was estimated at 91.9 %. f) Stored pollen samples kept for several days at ambient conditions is used to estimate the threshold (vertical line) between viable (right) and non-viable (left) pollen.


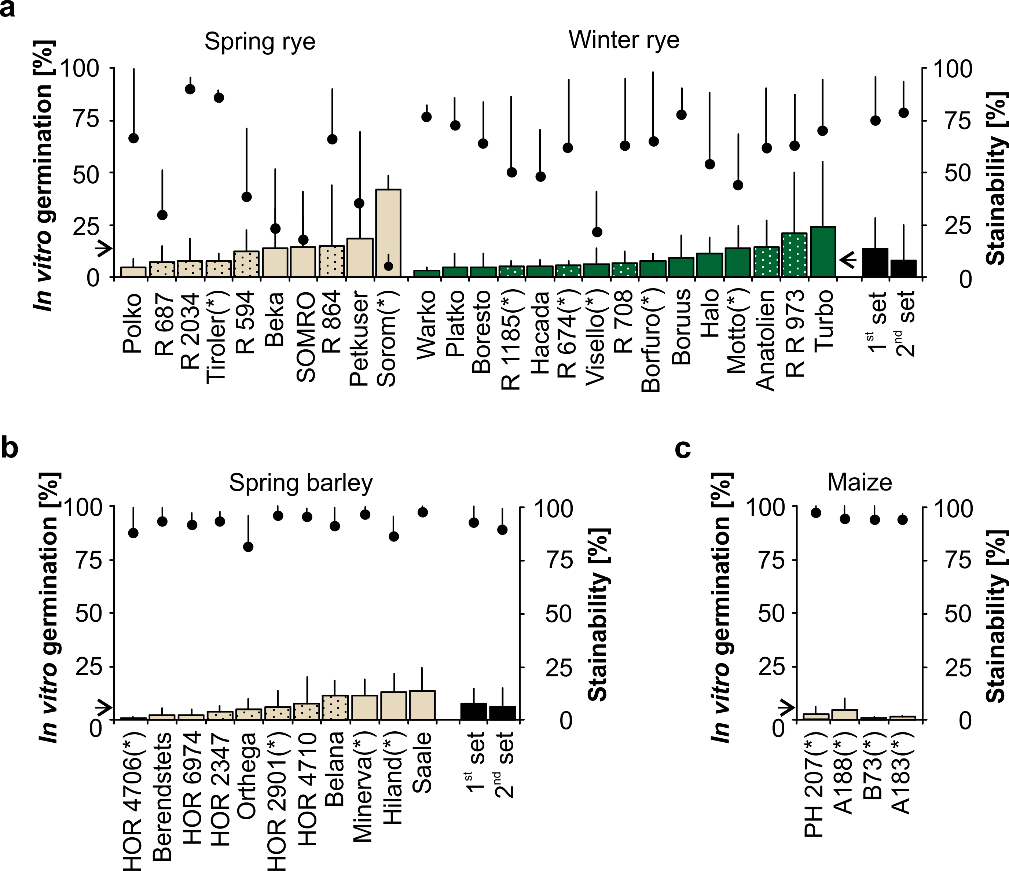


Supplementary Figure S3. *In vitro* pollen germination and viability varied between species of the Poaceae family and their lines. *In vitro* pollen germination (bars) was assessed on ‘Basic’ medium and compared to pollen stainability (points) assessed by FDA staining. Bars and points represent means and standard deviations of *in vitro* pollen germination of n = 3 (*) to 6 replicates of 100 to 300 pollen each and means and standard deviations of pollen stainability of n = 3 (*) to 6 replicates of 100 to 400 pollen, respectively. Arrows show the means of *in vitro* pollen germination for spring (left) and winter (right) lines. *In vitro* pollen germination (bars) and FDA staining was analyzed for a) 10 spring and 15 winter rye lines, among them 10 landraces (spotted bars) and 15 varieties (plain bars). Least significant difference at P < 0.05 (LSD5%) between lines was at 19.0 % for *in vitro* germination and at 32.3 % for pollen viability. Spring and winter rye differed significantly for pollen viability after FDA staining (LSD5% = 10.7 %). b) 11 spring barley lines, among them 6 landraces (spotted bars) and 5 varieties (plain bars) distinguish significantly for *in vitro* germination at LSD5% = 9.5 % and for pollen stainability at LSD5% = 10.5 %. c) *In vitro* germination and pollen stainability were not significantly different among the four maize lines.

# Supplementary Videos

Supplementary Video S1 Timelapse of anthesis shown within a wheat floret. Awns, glumes and lemmas were removed to observe swelling of the lodicules, elongation of the filaments, rupture of the anther tips and shedding of mature pollen on the stigma in a time interval of 8 minutes (161 images). At this stage, the maximum pollen maturity is reached and pollen was used for all experiments. The timelapse movie was created using the software NIS elements v. 4.11 (Nikon Metrology, Brighton, USA).
